# In *Hyphomicrobium denitrificans* Two Related Sulfane‐Sulfur Responsive Transcriptional Repressors Regulate Thiosulfate Oxidation and Have a Deep Impact on Nitrate Respiration and Anaerobic Biosyntheses

**DOI:** 10.1111/mmi.70002

**Published:** 2025-06-26

**Authors:** Jingjing Li, Nora E. Schmitte, Kaya Törkel, Christiane Dahl

**Affiliations:** ^1^ Institut für Mikrobiologie & Biotechnologie Rheinische Friedrich‐Wilhelms‐Universität Bonn Bonn Germany

**Keywords:** denitrification, *Hyphomicrobium denitrificans*, iron acquisition, PQQ, thiosulfate oxidation, transcriptional regulation, ubiquinone biosynthesis

## Abstract

Bacteria have evolved multiple strategies to sense and respond to the availability of inorganic reduced sulfur compounds such as thiosulfate. In *Hyphomicrobium denitrificans* , an obligately chemoorganoheterotrophic Alphaproteobacterium, the use of thiosulfate as a supplemental electron donor is regulated by two homologous sulfane‐sulfur‐responsive ArsR‐type transcriptional repressors, sHdrR and SoxR. Here, we provide information on the distribution and phylogeny of sHdrR, the relevance of its two conserved cysteines in vivo, and identify the genes controlled by SoxR and sHdrR not only by targeted qRT‐PCR but also by global RNA‐Seq‐based analyses of regulator‐deficient mutant strains. The absence of sHdrR and SoxR affected 165 and 170 genes, respectively, with 138 genes overlapping. SoxR regulates the *sox* genes for periplasmic thiosulfate oxidation and sulfane sulfur import into the cytoplasm, as well as the *lip‐shdr‐lbpA* genes encoding the cytoplasmic enzymes essential for sulfite formation. sHdrR affects only a subset of these genes. The transcription of *sox* genes remains unaltered in its absence. sHdrR and SoxR act cooperatively and their activity probably also involves interaction with other transcriptional regulators. Most importantly, sHdrR/SoxR regulation extends far beyond sulfur oxidation and deeply affects anaerobic metabolism, particularly denitrification in *H. denitrificans* .

## Graphical abstract

In *Hyphomicrobium denitrificans* , the use of thiosulfate as a supplemental electron donor is regulated by two homologous sulfane‐sulfur responsive ArsR‐type transcriptional repressors, sHdrR and SoxR, that act cooperatively. Remarkably, the regulation by sHdrR/SoxR extends far beyond sulfur oxidation and deeply affects anaerobic metabolism, particularly denitrification.

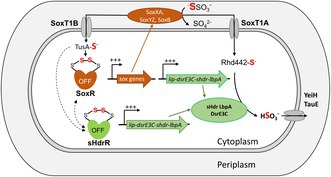

## 1 Introduction

Most dissimilatory sulfur‐oxidizing prokaryotes oxidize thiosulfate (S_2_O_3_^2−^), an inorganic sulfur compound of intermediary redox state. Accordingly, microbial thiosulfate oxidation has a major impact on global sulfur cycling. Thiosulfate oxidation under aerobic conditions is very widespread, particularly in marine environments (Podgorsek and Imhoff 1999; Marshall and Morris 2013; Watsuji et al. 2016). Anaerobic thiosulfate oxidizers have also been reported from a large range of environments, including anoxic marine basins (Menezes et al. 2020), oceanic oxygen minimum zones (Callbeck et al. 2021), and hydrothermal vents (Teske et al. 2000). The non‐toxic thiosulfate is readily available in such environments (Cardoso et al. 2006; Zhu and Getting 2012; Kumar et al. 2018) and it is likely that anaerobic oxidation, primarily through nitrate and nitrite reduction, is responsible for much of the thiosulfate removal (Ding et al. 2023). Thiosulfate‐dependent denitrification to N_2_ is best known for obligately autotrophic species such as *Thiobacillus denitrificans* , *Thiomicrospira denitrificans* , or *Sulfurovum lithotrophicum* (Inagaki et al. 2004), has been found to be important in marine chemosynthetic symbioses (Paredes et al. 2021), and has also been reported for the facultatively autotrophic *Paracoccus pantotrophus* (Robertson and Kuenen 1983).

Sulfur oxidizers include not only autotrophic prokaryotes from various groups, but also a large number of obligately organoheterotrophic bacteria that oxidize thiosulfate as an additional electron donor and are widely distributed in soil and natural waters (Trudinger 1967; Tuttle and Jannasch 1972; Sorokin et al. 1999; Ding et al. 2023). These include species of the genus *Hyphomicrobium*, Alphaproteobacteria that can be isolated from virtually any freshwater or soil sample, where they can constitute up to 0.2% of the total bacteria (Hirsch and Conti 1964; Gliesche et al. 2015; Li, Koch, et al. 2023). They are also prevalent in temporary puddles and in activated sludge, even under anaerobic conditions. Denitrifying hyphomicrobia such as *H. denitrificans* are of particular interest because of the need to remove nitrate in drinking water and sewage treatment plants, where these organisms are indeed highly abundant (Holm et al. 1996). *Hyphomicrobium* spp. are typically restricted to C_1_ and C_2_ compounds as carbon sources and are commonly identified as major players in denitrification systems supplied with methanol (Martineau et al. 2015). While enzymes required for denitrification have been purified and characterized from *H. denitrificans* (Deligeer et al. 2002; Yamaguchi et al. 2003, 2004), and genetic and physiological aspects of its denitrification are beginning to emerge (Meiberg et al. 1980; Martineau et al. 2015), these studies have not yet included any aspects of oxidative sulfur metabolism.

In *H. denitrificans* thiosulfate oxidation commences in the periplasm. Here, two thiosulfate molecules can be oxidatively linked to form the dead‐end product tetrathionate, a reaction catalyzed by thiosulfate dehydrogenase (TsdA) (Koch and Dahl 2018; Li, Koch, et al. 2023). Alternatively, thiosulfate can be completely oxidized to sulfate. Under aerobic conditions, this pathway is preferred at lower substrate concentrations (< 2.5 mM) and involves the periplasmic SoxYZ carrier protein to which thiosulfate is oxidatively bound by the action of the *c*‐type cytochrome SoxXA (Li, Koch, et al. 2023). Sulfate is then hydrolyzed off by SoxB and the sulfane sulfur remaining on SoxYZ is transferred to the cytoplasm via the membrane transporter SoxT1A (Li et al. 2024). Once inside, the sulfur is delivered through a cascade of sulfur transfer reactions to the sulfur‐oxidizing heterodisulfide‐reductase‐like enzyme complex, sHdr (Tanabe et al. 2024), which releases sulfite as a product. Sulfite is excreted and, in the absence of efficient sulfite‐oxidizing enzymes in *H. denitrificans* , chemically oxidized to sulfate in the presence of oxygen or may be transformed by other community members under environmental conditions (Li, Koch, et al. 2023; Li et al. 2024).

In the environment, *H. denitrificans* must not only cope with constantly changing concentrations of respiratory electron acceptors (oxygen, nitrate), but may also encounter varying concentrations of reduced sulfur compounds such as thiosulfate. To make the most of these additional electron sources, the organism must have strategies for sensing their presence. Indeed, we have recently identified two distinct but closely related ArsR‐SmtB family transcriptional repressors, SoxR and sHdrR, that are responsible for the transcriptional regulation of genes encoding Sox, sHdr and associated proteins in *H. denitrificans* (Li, Koch, et al. 2023; Li, Törkel, et al. 2023; Li et al. 2024). SoxR has been extensively characterized at the molecular level. Its sensing mechanism involves the formation of a sulfane sulfur bridge between two conserved cysteine residues in the presence of thiosulfate. In the sulfur‐bridged form, the repressor can no longer bind to its operator region(s) on the DNA and transcription is released (Li, Törkel, et al. 2023). Much less is known about sHdrR. It appears to regulate the sHdr system by modulating sHdrA levels, as seen in Western blot experiments, and to bind to the DNA region upstream of its own gene (Li, Koch, et al. 2023). Whether and how the two repressors interact in regulating the overall sulfur oxidation process and possibly other parts of hyphomicrobial energy conservation has not been studied in detail.

Here, we address these knowledge gaps by first providing insights into the relationship between SoxR and sHdrR, the distribution and phylogeny of sHdrR, and the relevance of the conserved sHdrR cysteines in vivo, and then collecting information on the SoxR and sHdrR regulons not only by targeted RT‐qPCR but also by a global RNA‐Seq‐based analysis of regulator‐deficient mutant strains, the latter revealing a profound effect on anaerobic metabolism, in particular denitrification.

## 2 Results and Discussion

### 2.1 Distribution and Phylogeny of sHdrR‐Related Proteins

**FIGURE 1:**
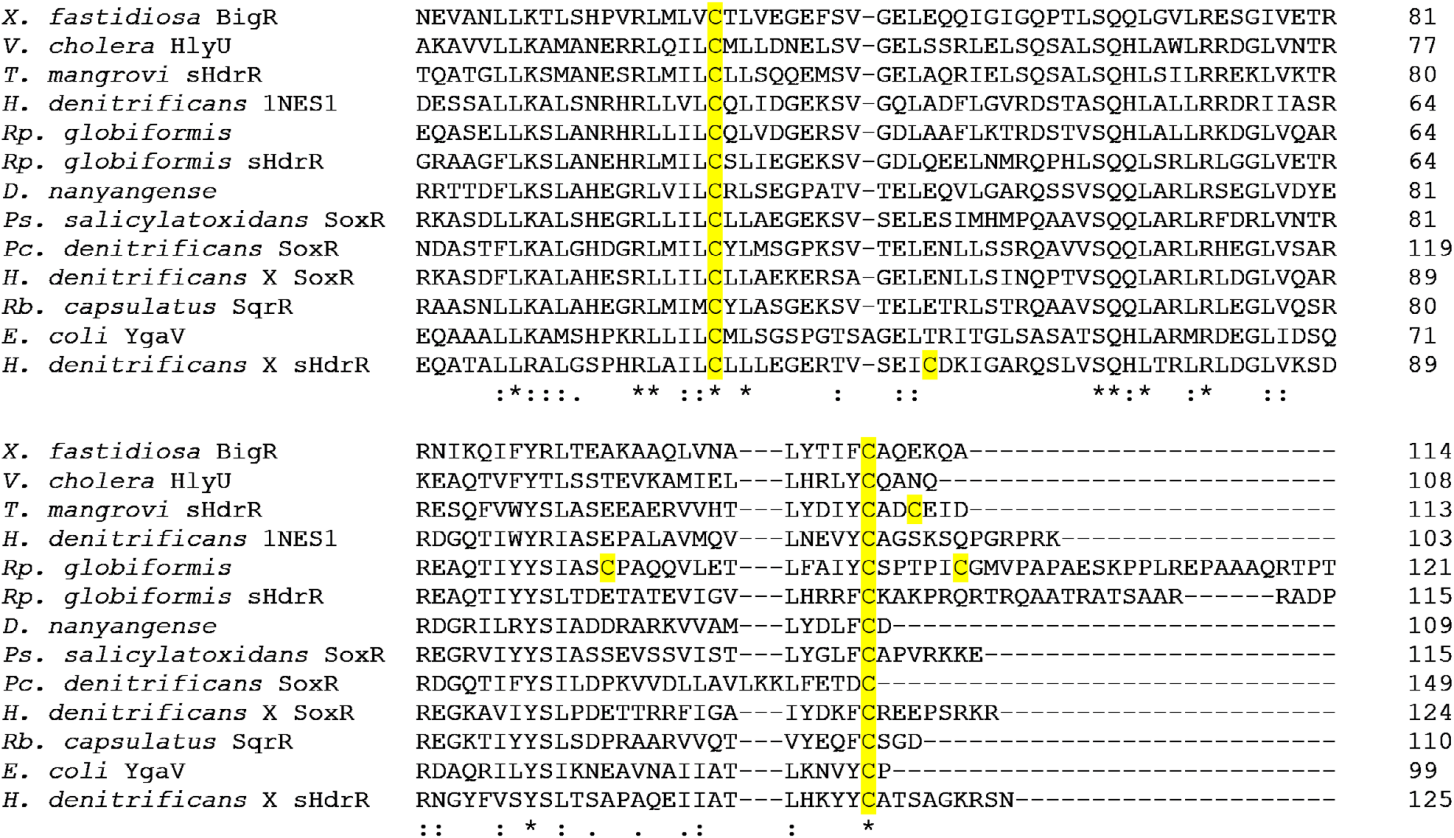
Amino sequence alignment of the central region of selected sHdrR homologs. A complete alignment is provided in Figure S1. Organism names, accession numbers/locus tags and references (if available) in the order of appearance: *Xylella fastidiosa* BigR, XF_0767 (Guimarães et al. 2011), *Vibrio cholera* HlyU, VC_A0642 (Mukherjee et al. 2014; Pis Diez et al. 2023), *Tsuneonella mangrovi*, CJO11_RS12710; *Hyphomicrobium denitrificans* 1NES1, HYPDE_25308 (Venkatramanan et al. 2013), *Rhodopila globiformis* , CCS01_RS26760 and *Rp. globiformis* sHdrR, CCS01_RS13140 (Imhoff et al. 2018), *Devosia nanyangense* HY834_20740 (He et al. 2021), *Pseudaminobacter salicylatoxidans* SoxR, WP_019171658 (Mandal et al. 2007), *Paracoccus denitrificans* SoxR, CAB94376 (Rother et al. 2005), *H. denitrificans* X^T^ SoxR, Hden_0700 (Li, Törkel, et al. 2023), *Rhodobacter capsulatus* SqrR, ADE85198 (Shimizu et al. 2017), *Escherichia coli* YgaV, b2667 (Gueuné et al. 2008), *H. denitrificans* X^T^ sHdrR, Hden_0682 (Li, Koch, et al. 2023; Li et al. 2024). Cysteines are highlighted in yellow. An * (asterisk) indicates positions with identical residues. Colons (:) and single dots (.) indicate conserved and semi‐conserved amino acids, respectively.

The *H. denitrificans* sHdrR (*Hd*sHdrR) protein is 125 amino acids in length, and a BlastP search identified *Rhodobacter capsulatus* SqrR (Shimizu et al. 2017) as the most similar structurally characterized protein. *Hd*sHdrR is also similar to several other characterized transcriptional regulators, all of which belong to the same ArsR subfamily and include SoxR from *Pseudoaminobacter salicylatoxidans* (Mandal et al. 2007), *Paracoccus denitrificans* (Rother et al. 2005) and *H. denitrificans* (Li, Törkel, et al. 2023), BigR from *Xylella fastidiosa* (Guimarães et al. 2011) and *Acinetobacter baumannii* (Walsh et al. 2020), YgaV from *Escherichia coli* (Gueuné et al. 2008; Balasubramanian et al. 2022) and HlyU from *Vibrio cholerae* (Pis Diez et al. 2023) (Figures 1, S1. All of these regulators share two conserved cysteine residues, numbered Cys^50^ and Cys^116^ in the hyphomicrobial protein (Figure 1). The characterized proteins sense reactive sulfur species (RSS) and form an intraprotomer tetrasulfide bridge between the conserved cysteines when exposed to sulfane sulfur transpersulfidation donors (Giedroc et al. 2023; Li, Törkel, et al. 2023).

In a previous publication, we performed a survey of the occurrence of genes for sHdrR‐like proteins based on an automatically generated hidden Markov model (HMM) with the additional condition that the respective gene is located in the vicinity of a *shdr*‐like gene cluster (Li et al. 2024). This revealed related transcriptional regulators in a wide variety of prokaryotes bearing the genetic potential for sulfur oxidation in the cytoplasm via the sHdr system. However, close examination of the amino acid sequences showed that many of the identified proteins do not contain the two conserved cysteine residues typical of sulfane‐sulfur‐responsive transcriptional repressors. This means not only that the HMM for sHdrR does not emphasize the conserved cysteines and must therefore be interpreted with caution, but also that the expression of *shdr* genes in many organisms may involve ArsR‐type regulators that rely on different modes of action.

In bacteria, transcriptional regulators often control the expression of genes located close to their own (Martinez‐Antonio and Collado‐Vides 2003; Browning and Busby 2004; Seshasayee et al. 2006). Therefore, we linked the proteins retrieved by a BlastP search using *Hd*sHdrR as a bait and the presence of the two conserved cysteines as a constraint with information about their genetic environment, and indeed we obtained revealing information (Table S1, Figure S2). The general picture that emerged from our analysis is that sulfane sulfur‐responsive ArsR‐type regulators appear to act not only on the expression of genes for enzymes involved in the oxidation and/or detoxification of sulfur compounds, such as *sqr* for sulfide: quinone oxidoreductase, *sox* for thiosulfate oxidation in the periplasm, *pdo* for persulfide dioxygenase, *rhd* for rhodanese‐type sulfur transferases, or *shdr* for sulfite production in the cytoplasm, but also on target genes encoding membrane proteins that have been shown or are likely to be involved in the transport of sulfur compounds across the cytoplasmic membrane, such as efflux pumps of the resistance‐nodulation‐division (RND) family (Nikaido 2011), sulfite exporters of the TauE family (Weinitschke et al. 2007), or SoxT and PmpAB, YeeE family transporters involved in the import of sulfur‐containing ions (Gristwood et al. 2011; Tanaka et al. 2020; Ikei et al. 2024; Li et al. 2024) (Figure S2, Table S1).

**FIGURE 2:**
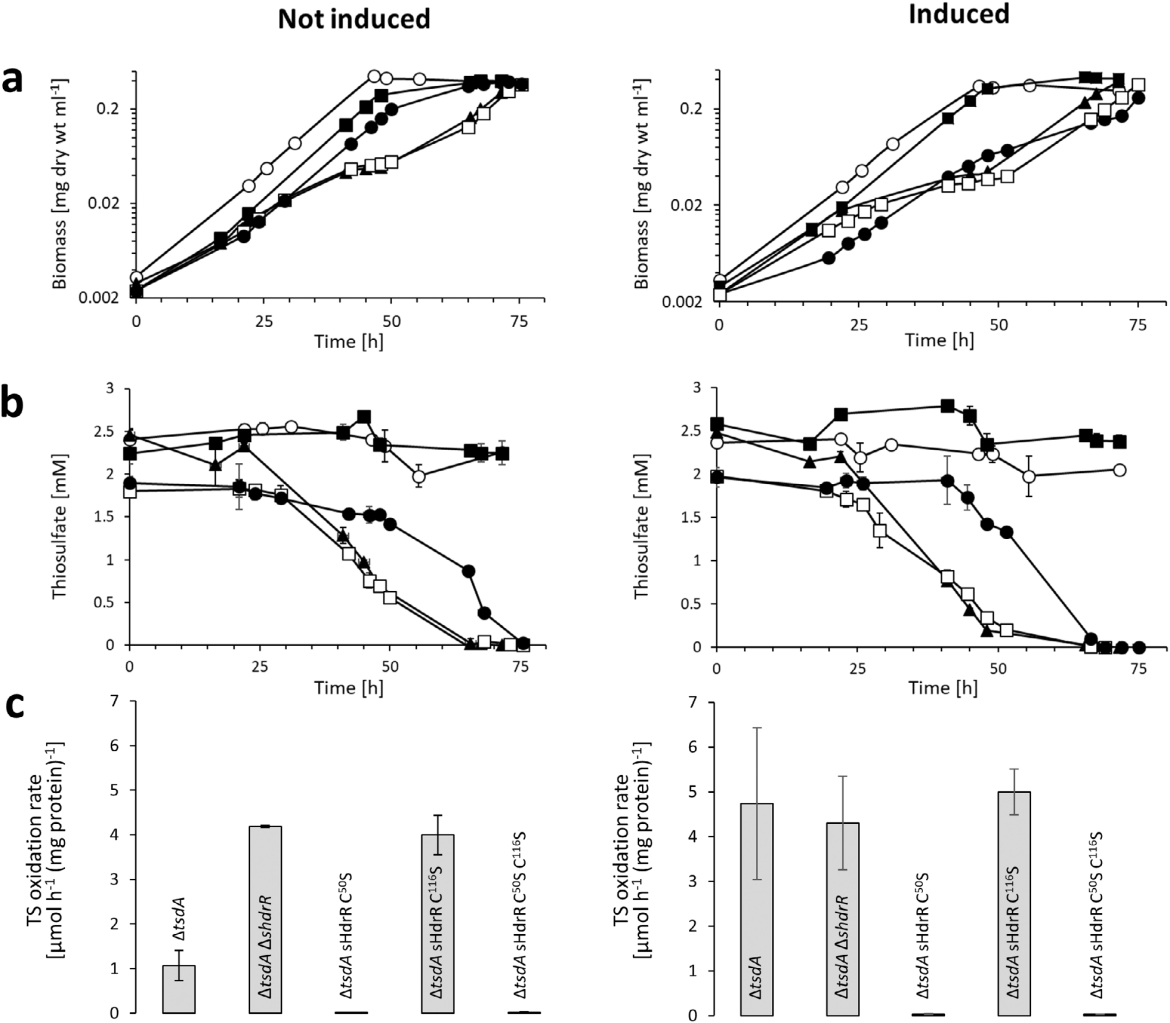
Growth and thiosulfate consumption of the *H. denitrificans* Δ*tsdA* reference strain, a strain lacking sHdrR and strains producing sHdrR variants with cysteine to serine exchanges. (a) *H. denitrificans* Δ*tsdA* (filled black circles), Δ*tsdA* Δ*shdrR* (open boxes), Δ*tsdA* sHdrR Cys^50^Ser (filled black boxes), Δ*tsdA* sHdrR Cys^116^Ser (filled black triangles) and Δ*tsdA* sHdrR Cys^50^Ser Cys^116^Ser (open circles). All strains were grown in medium containing 24.4 mM methanol. In the “not induced” case, pre‐cultures were grown without thiosulfate. In the “induced” case, pre‐cultures contained 2 mM thiosulfate. Error bars indicating SD are too small to be visible for determination of biomass. (b) Thiosulfate concentrations for the different cultures. Symbol assignments are the same as in the upper panels. (c) Specific thiosulfate oxidation rates derived from experiments shown in parts (a) and (b) were calculated as described in (Li, Koch, et al. 2023). All studied strains grew equally well on methanol in the absence of thiosulfate (Figure S3).

Remarkably, *Hd*sHdr‐related regulators with two conserved cysteines are rarely encoded in the vicinity of *shdr* genes (Table S1). A phylogenetic tree was calculated for sHdrR, SoxR and related transcriptional regulators. Although low bootstrap values suggest weak statistical support for certain branches, it is readily apparent that *Hd*sHdr‐related regulators encoded next to *shdrR* genes do not form a coherent phylogenetic clade (Figure S2). *Hd*sHdrR has a third cysteine, Cys^63^ (Figure 1). An equivalent of *Hd*sHdrR Cys^63^ is only present in a few cases (e.g., in the proteins from *Hyphomicrobium* sp. strains GJ21 and SCN 65–11). The third cysteine is absent in several regulators that are encoded in close proximity to *shdr* gene clusters (e.g., *Devosia nanyangense*, *Tsuneonella mangrovi*, and *Rhodopila globiformis* ) and on the other hand it is present in ArsR‐type proteins encoded in organisms such as the cyanobacterium *Sodalimona willei* and the Gram‐positive *Hathewaya proteolytica* that cannot oxidize sulfur compounds for dissimilatory purposes and do not contain *shdr* or *sox* genes (Figure S2, Table S1). It is therefore unlikely that this cysteine is of general importance.

### 2.2 Importance of Conserved sHdrR Cysteines In Vivo

In order to build a firm basis for the identification and characterization of sHdrR, we clarified whether the conserved cysteines (Cys^50^ and Cys^116^ in *Hd*sHdrR, Figure 1) are indeed necessary for the proper function of the regulator in vivo. This was achieved by phenotypic characterization of *H. denitrificans* mutant strains that contained chromosomal replacements of the wild‐type *shdrR* gene with variants encoding cysteine to serine exchanges of either one or both of the two conserved cysteines. All experiments reported in this and also previous studies (Li, Koch, et al. 2023; Li, Törkel, et al. 2023) were performed using *H. denitrificans* Δ*tsdA* as the reference strain and as the background strain for the introduction of further gene deletions or mutations. The Δ*tsdA* strain lacks the gene for thiosulfate dehydrogenase (TsdA). This enzyme catalyzes the oxidative formation of tetrathionate from two thiosulfate molecules. Thiosulfate dehydrogenase‐deficient strains are unable to form tetrathionate and thus are perfectly suited for analyzing the oxidation of the sulfur substrate to sulfite and eventually sulfate (Li, Koch, et al. 2023; Li, Törkel, et al. 2023; Li et al. 2024; Tanabe et al. 2023, 2024). The *H. denitrificans* Δ*tsdA* reference strain excretes sulfite as an intermediate en route to sulfate when methanol‐grown cultures are provided with thiosulfate as an additional electron source. Sulfite is toxic and leads to growth retardation that is particularly impressive when cultures are inoculated with thiosulfate‐induced cells ((Li, Koch, et al. 2023), also compare curves with filled circles in the upper panels of Figure 2). A Δ*tsdA* strain that additionally lacks the transcriptional repressor *shdrR* oxidizes thiosulfate without induction, produces sulfite instantaneously and, as a consequence, its growth rate slows down almost immediately when it is exposed to the sulfur compound ((Li, Koch, et al. 2023; Li et al. 2024), also compare curves with open boxes in the upper panels of Figure 2). When the original *shdrR* gene is re‐introduced into the chromosome, the wild‐type phenotype is reconstituted (Figure S4).

Like the *H. denitrificans* Δ*tsdA* Δ*shdrR* strain, the strain with sHdrR bearing a Cys^116^Ser exchange exhibited a significantly reduced growth rate even without induction of pre‐cultures (curves with filled triangles in Figure 2) and a high specific thiosulfate oxidation rate (Figure 2c). The growth rate increased significantly after thiosulfate was consumed. This appears to mean that in vivo the sHdrR Cys^116^Ser variant protein has a decreased DNA binding and thus transcription repressing activity. In contrast, both the mutant strain encoding the sHdrR Cys^50^Ser exchange and the mutant strain with both sHdrR cysteines replaced by serine were unable to oxidize thiosulfate (Figure 2c and curves with open circles and filled boxes in Figure 2a,b). The most obvious conclusion here would be that these sHdrR repressor variants constitutively repress transcription, that is, are always attached to their binding sites, and cannot react to the presence of oxidizable sulfur in vivo. A remaining obstacle is why the exchange of the two conserved sHdrR cysteines does not have the same effect. Both cysteines, at first glance, should be equally important for the formation of sulfur bridges between them. Detailed analysis of the related repressor SqrR from *R. capsulatus* provides a clue (Capdevila et al. 2021): Here, both relevant cysteines reside in a solvent accessible cavity, with one being significantly more solvent exposed, leading to reduced reactivity. This residue is probably not directly persulfidated but requires intramolecular sulfur donation from the other conserved cysteine. Similar differences in reactivity of the cysteines in the hyophomicrobial protein would well serve as a basis to explain the different observed phenotypes. In addition, we must take into account that the mutant strains studied still contain the fully functional SoxR regulatory protein, which we already know is the master regulator of sulfur oxidation in *H. denitrificans* (Li et al. 2024). Interactions of the sHdrR variants with this protein in vivo may complicate conclusions about their DNA binding capacity. Irrespective of this, our experiments clearly demonstrate the relevance of the two conserved cysteines.

### 2.3 Identification of Genes Controlled by sHdrR by RT‐qPCR for Different *H. denitrificans* Strains

**FIGURE 3:**
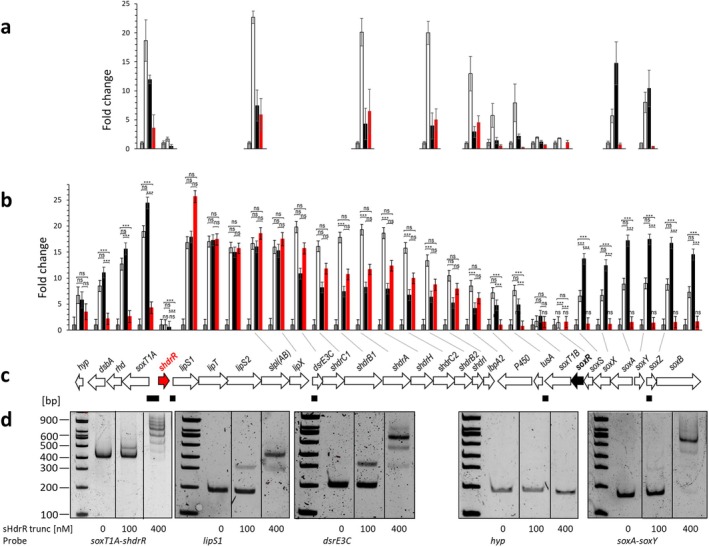
Regulation of the *shdr‐sox* region in *H. denitrificans* (a). Relative mRNA levels of 12 genes located in the *shdr‐sox* region (depicted in panel (c)) from *H. denitrificans* for the Δ*tsdA* reference strain in the absence (gray columns) and presence of thiosulfate (white columns), as assessed by RT‐qPCR. Results for *H. denitrificans* Δ*tsdA* Δ*soxR* and *H. denitrificans* Δ*tsdA* Δ*shdrR* in the absence of thiosulfate are shown by black and red columns, respectively. Results were adjusted using *H. denitrifcans rpoB*, which encodes the β‐subunit of RNA polymerase, as an endogenous reference according to (Martineau et al. 2015). All cultures were harvested in the exponential growth phase. Three parallel experiments were performed to obtain averages and standard deviation. (b) Transcript abundance changes of genes encoding enzymes involved in thiosulfate oxidation in the same strains as in (a) as assessed by RNA‐Seq analysis. The same color coding applies. The experiments were conducted in duplicate, each time using mRNA preparations from two different cultures. The standard errors of the changes were calculated using the delta approximation method (Taylor approximation). The adjusted *p* values for the statistically significant changes were all below 0.001 (Data S1), and are indicated by three asterisks (***p < 0.001). For clarity, asterisks were omitted in the high number of cases where there were significant differences between the mutant strains and the Δ*tsdA* reference strain in the absence of thiosulfate. ns, not significant (ns = (*p* ≥ 0.05). (c) DNA regions tested in EMSA assays for sHdrR binding are indicated as black rectangles below the hyphomicrobial *shdr‐sox* genetic island. Fragment sizes: 362 bp for the *soxT1A‐shdrR* intergenic region, 177 bp and 173 bp for the regions upstream of *lipS1* and *dsrE3C*, respectively. The fragments downstream of *tusA* and between *soxA* and *soxY* had sizes of 176 bp and 151 bp, respectively. (d) EMSA analysis of Strep‐tagged sHdr‐trunc with upstream promoter sequence probes of sulfur oxidation related genes as specified in (c). 17 nM DNA probes were incubated with different amounts of sHdr‐trunc (100 and 400 nM). Vertical lines separate samples that were run on the same gel but were not directly adjacent.

**FIGURE 4:**
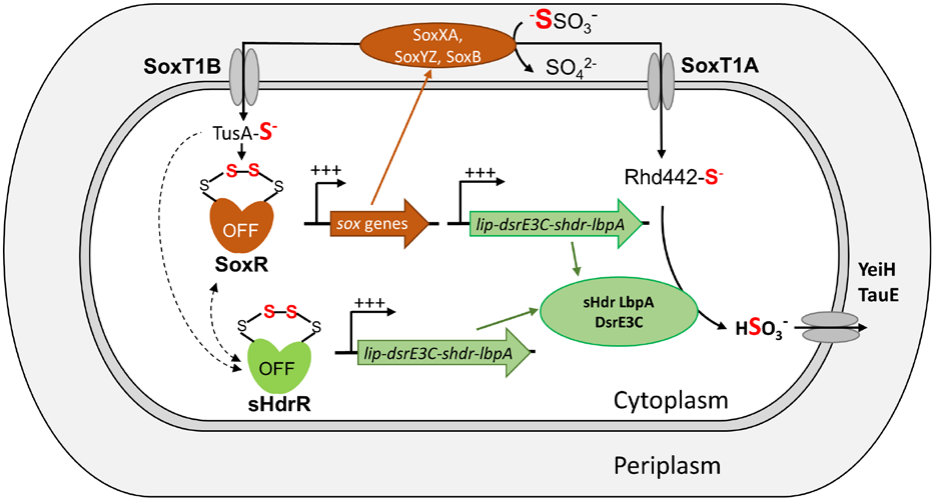
Proposed concerted function of SoxR and sHdrR in the regulation of sulfur metabolism in *H. denitrificans* . Thiosulfate oxidation is initiated in the periplasm, where SoxXA oxidatively attaches thiosulfate to SoxYZ and the sulfone sulfur is hydrolyzed off as sulfate by SoxB. The remaining sulfane sulfur is transported to the cytoplasm either by SoxT1B for signal transduction or by SoxT1A for its further oxidation by the sHdr proteins. The oxidation product sulfite is excreted by specialized transporters (YeiH (Hden_0834), TauE (Hden_0720), see Section 2.11). In the signaling pathway, sulfane sulfur becomes bound between two conserved cysteines in the regulators SoxR and sHdrR. It is not yet clear whether sulfur transfer from sulfur‐bridged SoxR to sHdrR can also occur. In their sulfur‐loaded form the repressor proteins can no longer bind to their target DNA and transcription sets in. SoxR controls the *sox*, as well as the *shdr* and associated genes. In contrast, sHdrR only affects a subset of these genes, which encode the enzymes involved in downstream oxidation of sulfane sulfur in the cytoplasm.

As a first step into the description of the sHdrR regulon, we performed RT‐qPCR and determined the transcription levels of 12 signature genes (Figure 3a) in the *H. denitrificans* sulfur oxidation locus (Figure 3c) for the Δ*tsdA* Δ*shdrR* mutant in the absence of thiosulfate and compared them with the *H. denitrificans* Δ*tsdA* reference strain. In addition to sHdrR, the signature genes encode two essential components of the periplasmic thiosulfate‐oxidizing Sox system (Li, Koch, et al. 2023), the SoxR repressor and the signal transducing membrane protein SoxT1B, two further proteins that may be involved in signal transduction (TusA and a cytochrome P450) (Li, Törkel, et al. 2023; Li et al. 2024), the sulfane sulfur importer SoxT1A (Li et al. 2024), three cytoplasmic proteins of the DsrE3C‐sHdr‐LbpA system responsible for sulfane sulfur transfer and its oxidation to sulfite (Cao et al. 2018; Koch and Dahl 2018; Tanabe et al. 2024), and Lpl(AB), one of the enzymes necessary for assembly of lipoate on the LbpA protein (Tanabe et al. 2023; Kümpel et al. 2024) (Figures 3 and 4). The absence of sHdrR led to constitutive transcription of genes *soxT1A*, *lipS1, shdrA*, and *shdrB2*, whereas the transcription of the other studied genes was not affected (Figure 3a). These results were fully confirmed by RNA‐Seq analysis (Figure 3b). We compared the RT‐qPCR and RNA‐Seq results for the *H. denitrificans* Δ*tsdA* Δ*shdrR* strain with the respective results for the Δ*tsdA* reference strain in the absence and presence of thiosulfate (Li, Törkel, et al. 2023), and those for a Δ*tsdA* Δ*soxR* mutant in the absence of thiosulfate (Figure 3b) (Li, Törkel, et al. 2023; Li et al. 2024) and found that sHdrR controls only a subset of the genes affected by the presence of thiosulfate or the lack of SoxR (Figure 3a,b). The sHdrR‐controlled subset does not include the genes of the divergently transcribed *soxYZ* and *soxAX* loci. The only genes whose transcription remained unaffected in the presence of the sulfur substrate or the lack of one repressor were those for the repressors themselves, and the signal‐transducing SoxT1B. A model for the concerted action of sHdrR and SoxR on the expression of genes relevant for sulfur oxidation is depicted in Figure 4.

The finding that sHdrR does not exert a significant effect on *sox* gene expression in vivo prompted us to analyze probable DNA binding regions. To that end, we inspected five intergenic regions within the hyphomicrobial sulfur oxidation locus in electrophoretic mobility shift assays (EMSA) with sHdrR (Figures 3c,d, S5). Four of the tested fragments contain prominent inverted or direct repeats (Figure S6). A fifth 176‐bp fragment served as a negative control. It is located upstream of Hden_0697, which encodes a putative cytochrome P450, and does not contain any conspicuous repeats. The EMSA experiments were performed with a carboxy‐terminally His‐tagged and amino‐terminally truncated version of sHdrR produced in *E. coli* that lacked the first 25 amino acids of the wild‐type protein. The truncation was necessary because the full‐length sHdrR proved to be very unstable, as documented by mass spectrometry, whereas the truncated variant could be used for a period of 7 days when kept on ice. The truncated sHdrR variant fully encompasses the central conserved region shown in Figure 1 and binds effectively to all but the control DNA probe (Figures 3d, S5). All four of the DNA fragments that were bound by sHdrR had previously been shown to contain binding sites for the closely related SoxR (Li, Koch, et al. 2023). Surprisingly, the intergenic region between the divergently oriented *soxA* and *soxY* genes is recognized by sHdrR in vitro (Figures 3d, S5), although a lack of sHdrR has no significant effect on the transcription of these genes (Figure 3a,b).

Given the close sequence similarity of SoxR and sHdrR, it is likely that their mechanism of action is similar, which would imply that the formation of a bridge of one to three sulfur atoms between the sulfur atoms of two conserved cysteine residues leads to a conformational change and unbinding of DNA (Figure 4) (Li, Törkel, et al. 2023). Whether and how the two transcriptional repressors compete for or interact at their binding sites in vivo cannot be answered on the basis of the available data. One possible model would be that SoxR and sHdrR co‐repress their target promoters by binding as two separate homodimers that have different binding affinities. An alternative model would be that these two proteins repress their target promoters as functional SoxR‐sHdrR heterodimers, although it is important to note that such interactions have only rarely been reported in bacteria (Kelm et al. 1997; Wehland and Bernhard 2000; Venkatesh et al. 2010; Al‐Bassam et al. 2014; Pannen et al. 2016).

### 2.4 Global Analysis of the sHdrR and SoxR Regulons by RNA‐Seq Analysis of Different *H. denitrificans* Strains

In the next step, we sought to identify the regulons of the intertwined transcriptional repressors SoxR and sHdrR on a global basis and extended previous genome‐wide mRNA‐Seq data for the Δ*tsdA* reference strain to *H. denitrificans* strains lacking either the genes for sHdrR or SoxR. We compared the mRNA abundance in these two strains to the mRNA abundance in the reference strain in the absence of thiosulfate. In addition, the data set for the reference strain in the presence of thiosulfate (Li et al. 2024) was integrated in the analyses. The number of identified mRNAs was virtually identical in all cases and ranged between 95 to 96% of the 3529 predicted genes.

**FIGURE 5:**
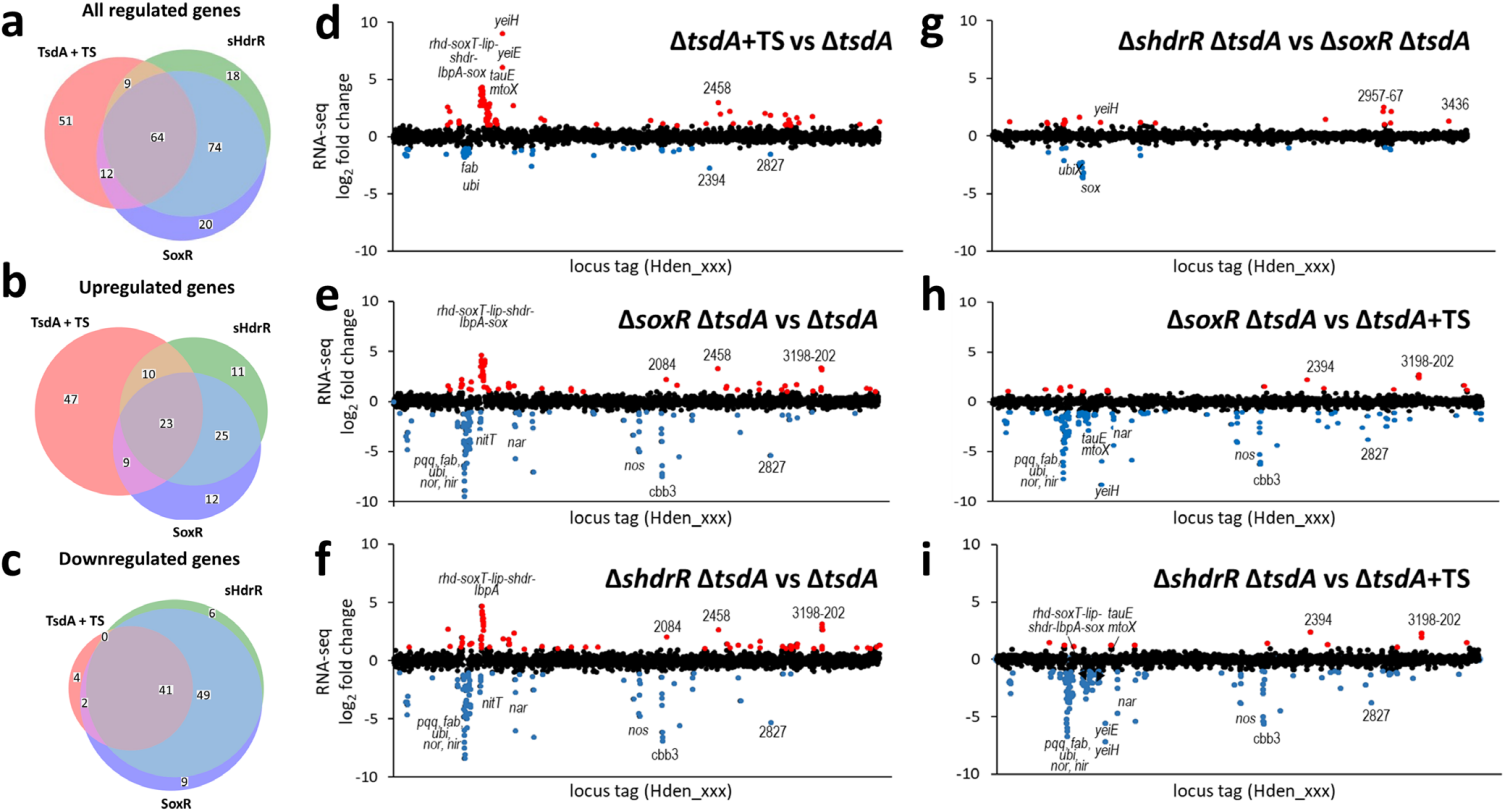
Global analysis of the sHdrR and SoxR regulons in *H denitrificans* by RNA‐Seq analysis. (a–c) Scaled Venn diagrams displaying the number differentially expressed genes from RNA‐Seq analysis of *H. denitrificans* strain Δ*tsdA* in the presence of thiosulfate (TsdA + TS, red), and strains Δ*tsdA* Δ*soxR* (SoxR, blue) and Δ*tsdA* Δ*shdrR* (sHdrR, green) in the absence of thiosulfate compared to *H. denitrificans* strain Δ*tsdA* in the absence of thiosulfate. The number of differentially expressed genes in each group is represented by the size of each circle, and overlapping areas indicate genes shared between the strains/conditions. Genes meeting a fold change of > 2 or < 0.5 and an adjusted *p*‐value of < 0.001 were considered differentially expressed. Numbers in each area are given for all regulated genes (a), all upregulated genes (b) and all downregulated genes (c). (d–i) Fold change in transcription for *H. denitrificans* Δ*tsdA* + thiosulfate (TS) versus Δ*tsdA* untreated (d), Δ*tsdA* Δ*soxR* untreated versus Δ*tsdA* untreated (e), Δ*tsdA* Δ*shdrR* untreated versus Δ*tsdA* untreated (f), Δ*shdrR* Δ*tsdA* versus Δ*tsdA* Δ*soxR* (g), Δ*tsdA* Δ*soxR* untreated versus Δ*tsdA* treated (h), *shdrR* Δ*tsdA* untreated versus Δ*tsdA* treated (i). Genes with a significant fold change (*p* < 0.001) in two biological replicates are highlighted (red for a > 2‐fold increase or blue for a < 0.5‐fold decrease).

Volcano plots (Figure S7) offer a comparative visual assessment of total gene expression patterns for each mutant relative to the wild type in the absence and in the presence of thiosulfate. The lack of the repressors sHdrR and SoxR affected the abundance of a total of 165 (4.8%) and 170 (5.0%) of the detected mRNAs, respectively, while the presence of thiosulfate affected the transcription of 136 (4.1%) genes in the reference strain. The genes affected by the lack of the two repressors exhibit a high overlap of 138 genes (83.6% and 81.2% of all genes affected by sHdrR and SoxR, respectively) (Figure 5a). Furthermore, 85 genes (65%) that are positively or negatively affected by the lack of either one or both of the repressors overlap with the genes affected by thiosulfate in the reference strain. We can therefore confidently state that SoxR and sHdrR are intimately involved in the cells' response to the availability of the oxidizable sulfur substrate thiosulfate.

Based on current models of how ArsR‐type repressors control gene expression (Busenlehner et al. 2003; Ren et al. 2017; Roy et al. 2018), removing the repressor proteins (i.e., deletion mutations as in the current study) should result in constitutive expression of genes that are normally repressed by that protein in the absence of the de‐repressing effector molecule. Decreased expression would indicate that the gene is activated (directly or indirectly) by the respective transcriptional regulator. Both positive and negative changes (fold changes > 2 or < 0.5, respectively) are observed for the SoxR and the sHdrR‐deficient mutant strains (Figure 5b,c). In the *H. denitrificans* Δ*tsdA* Δ*soxR* and Δ*tsdA* Δ*shdrR* strains, 69 genes are significantly overexpressed respectively, with an overlap of 69.6% of the genes (Figure 5b). In the Δ*tsdA* reference strain, the presence of thiosulfate positively influences the transcription of 89 genes, while a lower number of genes (47) is negatively affected (Li et al. 2024). The overlap with transcripts of higher abundance in the presence of thiosulfate in the reference strain is 37.1% and 36.0%, respectively (Figure 5b, Table S2). Conspicuously, there is a much higher overlap between the transcription of genes negatively affected by thiosulfate or the absence of either one of the repressors than seen for the genes with transcription increases (compare Figure 5b,c). While for the upregulated cases, 47 (52.8%) are induced by thiosulfate in the reference strain but do not show consequences upon deletion of the repressor genes, this is observed for only 4 (8.5%) of the downregulated cases. In addition, there are very few genes for which transcriptional downregulation is caused by the absence of only one of the regulators (Figure 5c). Only in 6.7% and 12.2% of cases, respectively, is the effect exclusively due to the absence of sHdrR or SoxR, while for genes with increased transcription the proportion amounts to 30.4% in both cases.

### 2.5 sHdrR and SoxR Regulate Thiosulfate Oxidation and Sulfate Assimilation

**FIGURE 6:**
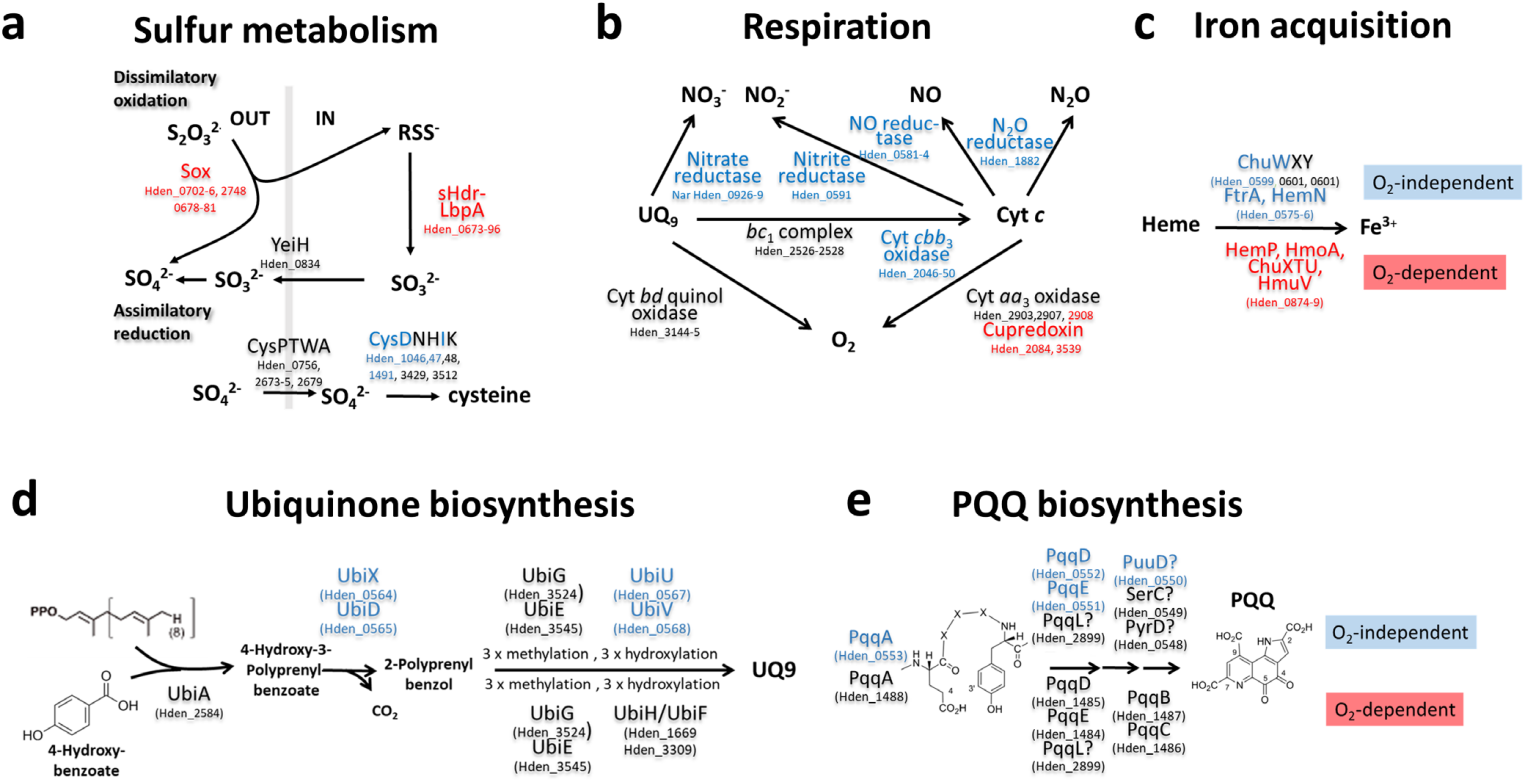
Effects of the absence of sHdrR or SoxR on central metabolic pathways in *H. denitrificans* X^T^. Proteins whose genes showed increased (> 2‐fold) transcript abundance either in *H. denitrificans* strain Δ*tsdA* Δ*shdrR* or in strain Δ*tsdA* Δ*soxR* or in both when compared to the *H. denitrificans* Δ*tsdA* reference strain in the absence of thiosulfate are printed in red. Proteins whose genes showed decreased (< 0.5‐fold) transcript abundance in either of the strains are printed in blue. Black letters indicate that significant changes in transcript abundance were not observed. (a) Dissimilatory oxidative and assimilatory reductive sulfur metabolism. (b) Electron delivery pathways to respiratory electron acceptors (c) Iron acquisition by heme degradation and iron release (d) Oxygen‐dependent and oxygen‐independent ubiquinone biosynthesis. In *E. coli*, ubiquinone biosynthesis starts from 4‐hydroxybenzoate, that is produced from chorismate (Abby et al. 2020), a step catalyzed by chorismate‐pyruvate lyase (UbiC) (Pelosi et al. 2016). We are currently unable to explain how 4‐hydroxybenzoic acid is synthesized in *H. denitrificans* and whether it is a mandatory precursor at all. *H. denitrificans* does not encode either a UbiC homolog or a homolog of XanB2, an unrelated chorismatase that fills the role of UbiC in *Xanthomonas campestris* (Zhou et al. 2013). In addition, it has been reported that alphaproteobacterial UbiA can accept *p*‐amino‐benzoic acid as a substrate for prenylation (Xie et al. 2015; Degli Esposti 2017). (e) (Proposed) pathways of PQQ synthesis in the absence or presence of oxygen.

Most of the genes whose transcription strongly increases in the absence of one or both of the two transcriptional regulators, as well as in the presence of thiosulfate in the reference strain, encode enzymes involved in oxidative sulfur metabolism (Figures 5d–f, 6a, Table S2). In full agreement with the RT‐qPCR analysis (Figure 3a), the transcription of the genes of the *soxT1A* operon, the *dsrE3C‐shdr‐lbpA* and the *lip* genes, which together encode proteins for sulfane sulfur import and its oxidation to sulfite, is strongly affected by the absence of either one, the sHdrR or the SoxR repressor (Figures 3b, 4, 5e,f). The situation is different for the *sox‐tusA‐p450* genes. Here, sHdrR deficiency has only a marginal effect, if any, which is again fully consistent with our RT‐qPCR results (Figures 3a,b, 4, 5f). The genes for the transcriptional repressors themselves and that for the signal transducing SoxT1B membrane protein are barely affected by thiosulfate or the absence of the other repressor.

*H. denitrificans* can use thiosulfate not only as a supplementary electron source but also as a sulfur source for cell constituents. Consistent with this, the transcription of genes involved in assimilatory sulfate reduction, such as those for sulfate adenylyltransferase CysDN, which activates sulfate to adenosine‐5′‐phosphosulfate, decreases in the repressor‐deficient *H. denitrificans* strains (Table S3, Figure 6a). The same was observed when the *H. denitrificans* Δ*tsdA* reference strain was exposed to thiosulfate (Li et al. 2024).

### 2.6 Impact of sHdrR and SoxR on Genes for Components of Aerobic Respiration and Anaerobic Respiration on Nitrate

In *H. denitrificans* , the absence of the repressors sHdrR or SoxR elicits responses that extend far beyond sulfur oxidation and assimilation, profoundly affecting central energy metabolism (Figures 5d–i, 6). A remarkable increase in transcript abundance, which occurs exclusively in the repressor‐deficient mutants, is observed for several genes encoding proteins involved in electron transport/aerobic respiration (Table S2, Figure 6b). Among these are Hden_2084 and Hden_3539, both of which encode periplasmic pseudoazurins/cupredoxins. While the small copper‐binding cupredoxins are best known as electron donors to the denitrification pathway (Kataoka et al. 2004; Impagliazzo et al. 2005; Fujita et al. 2012), members of the protein family can also be required for cytochrome *c* oxidase respiratory function under aerobic conditions (Castelle et al. 2010). On the other hand, transcript abundance of the genes for cytochrome *c* oxidase of type *cbb*_3_ is drastically reduced in sHdrR‐ or SoxR‐deficient mutant strains (Table S3, Figure 6b). This oxidase is adapted to low oxygen concentrations and plays a crucial role in microaerobic respiration (de Gier et al. 1996; Pitcher and Watmough 2004). It has a higher affinity to oxygen and is less efficient in proton pumping compared to the *aa*_3_‐type cytochrome *c* oxidase, the transcription of which remains unaffected. Furthermore, transcript abundance for virtually all genes underlying nitrate respiration is greatly reduced when either sHdr or SoxR are absent. Affected units include nitrate and nitrite transporters/antiporters (NitT, Nark), nitrate reductase (Nar), nitrite reductase (Nir), nitric oxide reductase (Nor), nitrous oxide reductase (Nos), electron carriers involved in denitrification and redox balance (cytochromes *c*, cupredoxin, NosR/NirI) as well as nitrate/nitrite and NO responsive regulators (NarQL, NnrS) (Figures 5e,f, 6b, Table S3).

It should be noted that the same differences are seen when the regulator‐deficient mutant strains are compared with the reference strain in the presence of thiosulfate (Figure 5h,i), that is, under conditions where neither of the two regulators can bind to its DNA target structures. Thus, a seemingly paradoxical phenomenon is observed: the simultaneous presence of two repressor proteins is imperative for maintaining normal transcription levels. This is probably not a direct effect but rather an indirect one exerted via the control of other transcription factors. One explanation would be that both repressors cooperate in the suppression of a stronger repressor. In their simultaneous presence, the transcription of target genes would be sustained at a normal level due to the absence of the stronger repressor. However, if the repression of the controlled repressor is compromised due to the absence of either SoxR or sHdrR, then larger amounts of the stronger repressor protein can be formed, potentially leading to significant repression of the transcription of target genes. Alternative explanations include participation in a feedback loop within a larger regulatory network, effects of SoxR/sHdrR on small regulatory RNAs, or the indirect joint influence of SoxR and sHdrR on the transcription of an activator protein. Our RNA‐Seq data point at the involvement of the latter possibility, as transcript abundance for the *narQL* genes (Hden_0557/0558), which encode a regulatory two‐component system, is drastically diminished in the SoxR‐ and sHdrR‐deficient strains (Table S3). In *E. coli* , the NarQ sensor responds to the presence of nitrate and/or nitrite by autophosphorylation followed by phosphoryl transfer to NarL, which results in its structural rearrangement and dimerization with subsequent DNA binding and promoter activation (Stewart 2003; Kompaniiets et al. 2024).

In 1999, Sorokin provided the first direct evidence of the ability of obligately heterotrophic bacteria to oxidize thiosulfate under anaerobic conditions (Sorokin et al. 1999). The organisms investigated in that study cannot oxidize thiosulfate completely to sulfate but form tetrathionate as an end product, while the *H. denitrificans* X^T^ wildtype strain can pursue both pathways. The reference strain we investigated here exclusively produces sulfate (Koch and Dahl 2018). The free energy released by reaction of thiosulfate with oxygen or nitrate is in a very similar range, with the process being slightly less favorable under anaerobic conditions (Equations 1 and 2), especially when considering the more reduced state of the respiratory chain in the absence of oxygen (Sorokin et al. 1999). The relatively small difference in free energy yield does not immediately appear as a convincing reason for strong regulatory integration of thiosulfate oxidation with aerobic and anaerobic respiration. It is also possible that the formation of toxic nitrite as an intermediate of denitrification reported for *H. denitrificans* X^T^ (Martineau et al. 2015) has an influence on overall regulation of respiratory processes in the organism.

(1) $$\begin{array}{c} S_{2}O_{3}^{2-}+2\;O_{2}+H_{2}O\to 2\;{\mathrm{SO}}_{4}^{2-}+2\;H^{+}\;\Delta G^{0\prime }-818\;\mathrm{kJ}\;{\mathrm{mol}}^{-1} \end{array}$$

(2) $$\begin{array}{c} S_{2}O_{3}^{2-}+1.6\;{\mathrm{NO}}_{3}^{-}+0.2\;H_{2}O\to 2\;{\mathrm{SO}}_{4}^{2-}+0.8\;N_{2}+H^{+}\;\Delta G^{0\prime }-766\;\mathrm{kJ}\;{\mathrm{mol}}^{-1} \end{array}$$

### 2.7 Impact of sHdrR and SoxR on Biosynthesis of Ubquinone (UQ_9_ )

In *H. denitrificans* , ubiquinone serves as an essential electron carrier in aerobic respiration on oxygen as well as in anaerobic respiration on nitrate. The organism contains only UQ_9_, with a side‐chain containing nine prenyl residues (Urakami and Komagata 1979, 1987). Menaquinone has not been detected in any *Hyphomicrobium* species. Accordingly, *H. denitrificans* lacks the menaquinone biosynthetic pathway established for *E. coli* as well as the alternative futasoline pathway (Dairi 2012).

Irrespective of the availability of oxygen, the first step in ubiquinone biosynthesis is prenylation of 4‐hydroxybenzoic acid by the membrane‐bound enzyme UbiA (in *H. denitrificans* : Hden_2584), followed by decarboxylation (catalyzed by the UbiX‐UbiD system) (Figure 6d). The pathway then requires three hydroxylations that are catalyzed by flavin‐dependent monooxygenases, UbiI, UbiF, and UbiH, in the presence of oxygen (Aussel et al. 2014). In *H. denitrificans* , two UbiH/UibF‐like monooxygenases (Hden_1669, Hden_3309) are suitable for the catalysis of these oxygen‐dependent reactions. Other proteobacteria also contain only two or even only one of these enzymes with relatively broad regioselectivity instead of the three specifically acting prototypical *E. coli* enzymes (Pelosi et al. 2016). In the absence of oxygen, the hydroxylations depending on molecular oxygen are replaced by oxygen‐independent steps catalyzed by the enzymes UbiT, UbiV, and UbiU (Pelosi et al. 2019), all of which are essential for denitrification in *Pseudomonas aeruginosa* (Vo et al. 2020). UQ biosynthesis further involves two *O*‐methylations (catalyzed by UbiG) and one *C*‐methylation (catalyzed by UbiE) (Figure 6d) (Aussel et al. 2014).

Our RNA‐Seq analysis reveals that oxygen‐independent UQ synthesis is co‐regulated with denitrification in *H. denitrificans* , with substantially diminished transcript abundance of an *ubiXDTUV* (Hden_0564 to 0568) gene set in the SoxR‐ and sHdrR‐deficient strains. In fact, *H. denitrificans* has only one copy each of *ubiD* and *ubiX*, and these are hardly transcribed in our sHdrR‐ and SoxR‐deficient mutant strains even when grown under full oxygen tension (Table S3, Figure 6d), suggesting that UbiD and UbiX may be replaced by so far unidentified non‐orthologous proteins under aerobic conditions. The existence of alternative decarboxylases is supported by the fact that some ubiquinone‐containing Alphaproteobacteria lack *ubiD* and *ubiX* completely. In addition, the decarboxylating enzyme has not yet been identified in mitochondria (Guerra and Pagliarini 2023).

### 2.8 Impact of sHdrR and SoxR on Fatty Acid Biosynthesis

The genes for oxygen‐independent UQ_9_ biosynthesis and nitric oxide reductase plus accessory and regulatory proteins are clustered in *H. denitrificans* (Hden_0564 to Hden_0598) and are immediately preceded by another gene set, Hden_0554 to Hden_0563, whose transcript abundance decreases significantly in the absence of either SoxR or sHdrR (Table S3). The encoded proteins all appear to be related to fatty acid biosynthesis and include four genes for 3‐oxoacyl‐ACP‐[acyl‐carrier protein]‐synthase II or possibly 3‐oxoacyl‐ACP‐synthase I, FabF, one each for FabG (3‐oxoacyl‐ACP reductase) and FabZ (beta‐hydroxyacyl‐ACP dehydratase) and two for acyl carrier proteins (Hden_0560, 0563). For all of these genes, *H. denitrificans* has at least one additional copy residing somewhere else on the genome. The last step of the elongation cycle during fatty acid synthesis is catalyzed by enoyl‐ACP reductase (FabI). However, the canonical hyphomicrobial FabI enzyme, Hden_1970, which is a member of the short‐chain dehydrogenase/reductase superfamily does not have a counterpart in the gene cluster underlying SoxR/sHdrR control. We speculate that the product of Hden_0556, annotated as alcohol dehydrogenase, performs this function under anaerobic/denitrifying conditions. It is well known that some bacterial species contain additional enoyl‐ACP reductases (Massengo‐Tiasse and Cronan 2009; Hopf et al. 2022). In summary, we suggest that the mentioned gene products work together in a fatty acid biosynthesis pathway that is especially efficient in/designed for anaerobic/denitrifying conditions.

### 2.9 Impact of sHdrR and SoxR on Biosynthesis of PQQ

The genes Hden_0551–0553, which are located next to the genes probably involved in fatty acid biosynthesis in the absence of oxygen, are also transcribed at a much lower level in the SoxR and sHdrR‐deficient *H. denitrificans* mutants than in the reference strain (Table S3, Figure 6e). The encoded proteins are involved in the biosynthesis of pyrroloquinoline quinone (PQQ). PQQ is a cofactor of periplasmic quinoprotein dehydrogenases such as cytochrome *c‐*dependent methanol dehydrogenase, an enzyme of major importance to *H. denitrificans* when it grows on methanol (Duine and Frank Jr. 1980a, 1980b; Dijkstra et al. 1989). PQQ is synthesized from a precursor peptide, PqqA, with the conserved sequence motif E‐X_3_‐Y (Cordell and Daley 2022) (Figure 6e). PqqA is bound by PqqD (Latham et al. 2015), and bond formation between the glutamate and tyrosine C9 atoms is catalyzed by PqqE. The next step involves the cleavage of the structure from PqqA, which is catalyzed by PqqF/PqqG/PqqH and/or other proteases (Cordell and Daley 2022). PqqB probably hydroxylates and oxidizes the Glu‐Tyr dipeptide yielding 3a‐(2‐amino‐2‐carboxyethyl)‐4,5‐dioxo‐4,5,6,7,8,9‐hexahydroquinoline‐7,9‐di‐carboxylic acid (AHQQ). The last step is ring cyclization and eight‐electron oxidation catalyzed by PqqC. The reaction includes four oxidative steps requiring molecular O_2_ and hydrogen peroxide (H_2_O_2_) (Bonnot et al. 2013). *H. denitrificans* X^T^ contains the full gene set for PQQ synthesis under aerobic conditions (PqqABCDE, Hden_1484–1488). Genes for PqqF/G/H are not present, but their function could be taken over by PqqL (Hden_2899) or other peptidases (Grinter et al. 2019; Cordell and Daley 2022). Second copies for *pqqA*, *pqqD*, and *pqqE* are located in the gene cluster that is under SoxR/sHdrR control (Hden_0552 to 0553), and it is tempting to speculate that the final steps of PQQ biosynthesis in the absence of oxygen may be encoded in the same transcriptional unit and undergo the same transcriptional regulation. There is some circumstantial support for this suggestion: The products of Hden_0550 and Hden_0548 are similar to the N‐terminal domain with four transmembrane helices of cytochrome *c* urate oxidase, PuuD (Doniselli et al. 2015), and to dihydroorotate oxidase, PyrD (Larsen and Jensen 1985), respectively, both of which catalyze reactions on carbon‐ and nitrogen‐containing heterocycles with certain structural similarities to precursors of PQQ. Hden_0549 encodes a putative phosphoserine aminotransferase, SerC (Duncan and Coggins 1986). Clusters of a *pqqAED‐puuD‐serC‐pyrD* are not only present in addition to classical *pqqABCDE* clusters in other *Hyphomicrobium* species, for example, *H. nitrativorans*, but also in the Gammaproteobacteria *Halomonas sulfidovorans* strain MCCC 1A13718 (locus tags for the two *pqqE* genes: HNO53_16555 and HNO53_16620), *Stutzerimonas* (former *Pseudomonas*) *stutzeri* (*pqqE* genes in strain CGMCC 22915: NPN27_09805 and NPN27_22,915) and *Methylophaga nitratireducenticrescens* JAM1 (*pqqE* genes: Q7A_453 and Q7A_868), and the Betaproteobacterium *Azoarcus* sp. DN11 (*pqqE* genes: CDA09_04515 and CDA09_08755). All of these organisms are capable of a denitrifying metabolism (Kasai et al. 2007; García‐Valdés et al. 2010; Martineau et al. 2013; Wang and Shao 2021) and may therefore be prepared for PQQ synthesis under these conditions. *Klebsiella pneumonia* and *Pseudomonas aeruginosa* are counterexamples. They contain only the canonical *pqqABCDEF/H* genes, and PQQ is not synthesized during anaerobic growth, although the *pqq* gene set is transcribed (Velterop et al. 1995; Gliese et al. 2010).

### 2.10 sHdrR and SoxR Play Roles in Iron Acquisition Under Aerobic and Anaerobic Conditions

When we look at further genes with altered transcript abundance only in the regulator‐deficient mutants, several sets of functionally coherent genes stand out (Figures 5d–f, 6c, Table S2,S3). The encoded enzymes all play a role in iron acquisition via uptake and subsequent degradation of hemin (Wandersman and Delepelaire 2004). The genes for two putative oxygen‐dependent heme‐degrading HmoA monoxygenases (Frankenberg‐Dinkel 2004), Hden_0541 and Hden_0875, are more highly transcribed in the mutants strains. Key subunits of the sulfur‐oxidizing Sox and sHdr systems contain either heme or iron–sulfur clusters, and it is therefore not surprising that cells prepare for thiosulfate oxidation by taking steps to ensure that these enzymes are equipped with the necessary prosthetic groups.

On the other hand, strong decreases in transcript abundance are observed in the sHdrR and SoxR‐deficient mutant strains for genes encoding proteins needed for heme degradation and iron acquisition under anaerobic conditions (Figures 5d–f and 6c, Table S3). FtrA (Hden_0575) is a periplasmic iron protein, HemN (Hden_0576) serves as an oxygen‐independent coproporphyrinogen III oxidase (Layer et al. 2002) and ChuW (Hden_0599) is a radical S‐adenosylmethionine methyltransferase that catalyzes a radical‐mediated mechanism facilitating iron liberation and the production of a tetrapyrrole product called “anaerobilin” (LaMattina et al. 2016). It serves as a substrate for ChuY, an anaerobilin reductase (Hden_0601), possibly also involving ChuX, a putative heme binding protein (Hden_0600). The transcript abundance for these two genes is not affected in the repressor‐negative mutants.

The drastic changes in the transcription of genes for proteins responsible for iron availability under aerobic versus anaerobic conditions, in the absence of either repressor protein, highlight the importance of cooperative action between both regulators to maintain optimal transcription levels of relevant genes. Comparable increases in abundance are not triggered by thiosulfate in the reference strain if it grows on the same defined medium with sufficient trace elements. It is thus evident that mutants lacking a single repressor are incapable of responding to iron availability. A logical explanation for this phenomenon is that the two repressors, functioning in conjunction, control the production of an iron‐responsive regulator.

### 2.11 RNA‐Seq Analysis Reveals Transcriptional Regulation Induced by Thiosulfate, but Independent of sHdrR and SoxR

The two genes that show the highest abundance changes (526‐ and 66‐fold, respectively) in the reference strain upon addition of thiosulfate, encode a sulfite exporter (Hden_0834, YeiH) and a LysR family transcriptional regulator (Hden_0835) (Li et al. 2024). Surprisingly, the transcription of these two genes is not affected by the removal of sHdrR or SoxR. Closer inspection of the LysR‐type protein revealed that its most closely related structurally and biochemically characterized homologs are NdhR (or CcmR) from *Synechocystis* PCC6803 (5Y2V (Jiang et al. 2018)) and YeiE from *Cronobacter sakazaki* (7ERQ_A (Hong et al. 2022)). While 2‐phosphoglycolate is an inducer for NdhR, YeiE serves as a global virulence regulator in *C. sakazakii* , binds sulfite as an effector and has a central role in defending against sulfite toxicity (Hong et al. 2022). The Hden_0835 derived protein shares five of seven sulfite‐interacting residues with *C. sakazakii* YeiE. Notably, these include Glu^145^, which is responsible for discriminating between sulfite and sulfate (Figure S8). In contrast, of the seven residues in NdhR that interact with 2‐phosphoglycolate, only three are present in the *H. denitrificans* protein. Toxic sulfite is formed as an intermediate during thiosulfate oxidation by *H. denitrificans* (Li, Koch, et al. 2023) and is effectively excreted into the medium, probably mainly by the action of the YeiH exporter (Li et al. 2024). It is likely that the YeiE protein derived from Hden_0835‐recognizes sulfite and activates transcription of the neighboring *yeiH* gene upon binding of the inducer. This is only seen in the *H. denitrificans* Δ*tsdA* reference strain because the sHdrR‐ and SoxR‐deficient strains were cultivated in the absence of thiosulfate.

There is another set of genes, Hden_0719 to Hden_0748, for which drastic changes in abundance occur when the reference strain is exposed to thiosulfate, but whose transcription is not triggered by the absence of sHdrR or SoxR, that is, these genes do not belong to the regulons of the two repressors. The affected genes include those for a putative dimethylsulfide (DMS) monooxygenase and for methanethiol oxidase, MtoX (Eyice et al. 2017). In *H. denitrificans* thiosulfate is formed during DMS degradation, which occurs with methanethiol as an intermediate product (Koch and Dahl 2018). We conclude that cells that sense thiosulfate are stimulated to prepare for degradation of the environmentally abundant organosulfur compound.

## 3 Conclusions

The hyphomicrobial ArsR‐type regulator proteins sHdrR and SoxR belong to the same family of sulfane‐sensitive transcriptional repressors, which are characterized by two conserved and essential cysteines. Both proteins are directly involved in controlling sulfur oxidation, but sHdrR regulates only a subset of SoxR‐dependent genes (Figure 4). Surprisingly, our work expands the role of the sHdrR/SoxR regulatory system far beyond sulfur oxidation and reveals a large group of genes whose transcription is drastically reduced by the absence of either repressor (Figures 5 and 6, Table S3). Virtually all of the products of these genes share one overarching feature: they are involved in anaerobic metabolic pathways, particularly in energy conservation in the absence of oxygen. It is well known that *H. denitrificans* can grow using methanol as a source of carbon and electrons with respiration using nitrate as an electron acceptor. During the aerobic growth of our regulator‐deficient mutants, we observed a negative effect on the transcription of all components involved, ranging from enzymes that drive denitrification to anaerobic synthesis of the electron carrier ubiquinone and O_2_–independent synthesis of the cofactor PQQ, which is essential for methanol dehydrogenase. The negative effect observed upon the loss of sHdrR and SoxR is not an expected consequence of repressor deletion. sHdrR and SoxR could act as direct activators, but we consider it more likely that the negative effect is indirect, and that they are repressors of other negatively acting transcription factors. Regardless of whether the negative effects exerted by the loss of sHdrR and SoxR are direct or indirect, our analyses revealed that the regulation of sulfur oxidation and anaerobic respiration are deeply intertwined. To our knowledge, this aspect has not yet been reported for chemoorganoheterotrophic sulfur oxidizers, and it has only become apparent through our investigation of *H. denitrificans* mutant strains. The present study has set the stage for future research, which will further elucidate the intricate relationship between oxidative sulfur metabolism and denitrification. Enhanced understanding of these processes promises significant insights into the biology of these bacteria, particularly their role in environmental contexts, such as their contribution to greenhouse gas emissions (e.g., N_2_O).

## 4 Experimental Procedures

### 4.1 Bacterial Strains, Plasmids, Primers, and Growth Conditions

*H. denitrificans* strains were cultured in minimal medium kept at pH 7.2 with 100 mM 3‐(*N*‐Morpholino)propanesulfonic acid (MOPS) buffer as previously described (Koch and Dahl 2018). Media contained 24.4 mM methanol. Thiosulfate was added as needed. *E. coli* strains were grown on complex lysogeny broth (LB) medium (Bertani 2004) under aerobic conditions at 37°C unless otherwise indicated. *Escherichia coli* BL21 (DE3) was used for recombinant protein production. *E. coli* strains 10‐beta and DH5α were used for molecular cloning. Antibiotics for *E. coli* and *H. denitrificans* were used at the following concentrations (in μg mL^−1^): ampicillin, 100; kanamycin, 50; streptomycin, 200; chloramphenicol, 25. Table S4 lists the bacterial strains and plasmids that were used for this study.

### 4.2 Recombinant DNA Techniques

Restriction enzymes, T4 ligase and Q5 polymerase were obtained from New England Biolabs (Ipswich, UK) and used according to the manufacturer's instructions. Oligonucleotides were obtained from Eurofins Genomics Germany GmbH (Ebersberg, Germany). Standard techniques for DNA manipulation and cloning were used unless otherwise indicated (Ausubel et al. 1997). Plasmid DNA from *E. coli* was purified using the GenJET Plasmid Miniprep kit (Thermo Scientific, Waltham, USA). Chromosomal DNA from *H. denitrificans* strains was prepared using the Simplex Easy DNA Extract Kit (GEN‐IAL GmbH, Troisdorf, Germany). DNA fragments were extracted from agarose gels using the GeneJET Gel Extraction Kit (Thermo Scientific, Waltham, USA).

### 4.3 Overproduction and Purification of Recombinant Truncated sHdrR

A truncated version of the *H. denitrificans shdrR* gene not encoding the first 25 amino acids of the wildtype protein was amplified from *H. denitrificans* genomic DNA using primers Fr‐pET22b‐sHdrR‐trunc‐NdeI and Rev‐pET22b‐0682‐NotI and (Table S4) and cloned between the NdeI and NotI sites of pET22b (+), resulting in pET22bHdsHdrR‐trunc. Recombinant sHdrR was overproduced in *E. coli* BL21 (DE3). The cells were grown at 37°C in 200 mL LB medium containing ampicillin up to an OD_600_ of 0.6. Expression of *shdrR‐trunc* was induced by adding 0.5 mM IPTG. IPTG‐induced *E. coli* cells were grown over night at 20°C. The carboxy‐terminally His‐tagged protein was purified by affinity chromatography on Ni^2+^‐NTA using the same conditions as described for the full length protein (Li, Koch, et al. 2023).

### 4.4 Construction of *H. denitrificans* Mutant Strains

The suicide plasmid pk18*mobsacB* (Schäfer et al. 1994) and the tetracycline cassette from pHP45Ω‐Tc (Fellay et al. 1987) were used for reverse genetics in *H. denitrificans* . Derivatives were constructed on the basis of previously published procedures (Cao et al. 2018; Koch and Dahl 2018). For chromosomal complementation of the *H. denitrificans* Δ*tsdA* Δ*shdrR* strain, the *shdrR* gene was amplified together with upstream and downstream regions using primers Fwd_deltaHden0682_BamHI and Rev_deltaHden0682_XbaI and cloned into the XbaI/BamHI sites of pk18*mobsacB*. For chromosomal integration of the genes encoding sHdrR Cys^50^Ser, sHdrR Cys^116^Ser, and sHdrR Cys^50^Ser Cys^116^Ser, the modified genes and upstream and downstream sequences were amplified by SOE PCR using the appropriate primers listed in Table S4. Finally, the tetracycline resistance cassette from pHP45ΩTc was inserted into each of the plasmids using SmaI. The final constructs were electroporated into *H. denitrificans* Δ*tsdA* Δ*shdrR* and transformants were selected using previously published procedures (Cao et al. 2018; Koch and Dahl 2018). Single crossover recombinants were Cm^r^ and Tc^r^. Double crossover recombinants were Tc^s^ and survived in the presence of sucrose due to the loss of both the vector‐encoded levansucrase (SacB) and the tetracycline resistance gene. The genotype of the *H. denitrificans* strains generated in this study was confirmed by PCR and sequencing.

### 4.5 Characterization of Phenotypes, Quantification of Sulfur Compounds and Biomass Content

Growth experiments with *H. denitrificans* were run in medium with 24.4 mM methanol in Erlenmeyer flasks as described earlier (Li, Koch, et al. 2023). 2 mM thiosulfate was added when needed. Biomass content, thiosulfate, and sulfite concentrations were determined by previously described methods (Dahl 1996; Li, Koch, et al. 2023). All growth experiments were repeated three to five times. Representative experiments with two biological replicates for each strain are shown. All quantifications are based on at least three technical replicates.

### 4.6 Electrophoretic Mobility Shift Assays (EMSA)

The binding reaction mixtures for EMSA assays (15 μL final volume) contained purified truncated sHdrR protein in various concentrations (up to 400 nM), 2 μL 50% glycerol, and 1.5 μL 10× binding buffer (100 mM Tris–HCl, 500 mM KCl, 10 mM DTT, 5% glycerol, pH 8.0). Reaction mixtures were pre‐incubated for 20 min at room temperature, followed by a 30 min incubation at 30°C after adding the DNA probe to a final concentration of 17 nM. DNA probes were prepared using the primers listed in Table S4. The DNA probes were the same as described in (Li, Törkel, et al. 2023). After pre‐running 6% native polyacrylamide gels at 100 V for 1 h at 4°C with 0.25× TBE buffer (25 mM Tris‐borate, 0.5 mM EDTA), they were loaded with the reaction mixtures. 0.25× TBE with 0.5% glycerol was used as running buffer. The gels were run at 180 V for 1 h at 4°C. Gels were subsequently stained for 20 min with SYBR green I. The bands corresponding to sHdrR‐bound and free DNAs were visualized with a ChemiDoc Imaging System (BioRad).

### 4.7 Expression Studies Based on RT‐qPCR

Total RNA of the relevant *H. denitrificans* strains was isolated from cells harvested in mid‐log phase according to an established procedure (Li, Törkel, et al. 2023). RNA samples of 100 ng were used for RT‐qPCR analysis, which was performed with the primers listed in Table S4 following the method described in (Li, Törkel, et al. 2023).

### 4.8 Genome‐Wide Transcriptomic Analysis of *H. denitrificans* Strains Δ*tsdA* Δ*shdrR* and Δ*tsdA* Δ*soxR* in the Absence of Thiosulfate

For transcriptome sequencing (RNA‐Seq), *H. denitrificans* strains Δ*tsdA* Δ*shdrR* and Δ*tsdA* Δ*soxR* were cultured in 50 mL minimal medium containing 24.4 mM methanol in 200 mL Erlenmeyer flasks at 30°C with shaking at 200 rpm to early log phase. The experiments were conducted in duplicate, each time using mRNA preparations from two different cultures. Cells from a 20 mL culture were harvested and flash frozen in liquid N_2_ and stored at −70°C. As described in Li et al. (2024), the RNA was purified with the FastGene RNA Premium Kit (NIPPON Genetics Europe, Düren, Germany) according to the manufacturer's instructions and with the introduction of a modified cell lysis step by bead beating. RNA quality was checked on 1% agarose gels and its concentration was measured using NanoPhotometer NP80 (IMPLEN, Munich, Germany). The RNA was shipped on dry ice to Eurofins Genomics GmbH (Ebersberg, Germany). The subsequent analysis pipeline included rRNA depletion, library preparation (mRNA fragmentation, strand specific cDNA synthesis), Illumina paired‐end sequencing (2 × 150 bp, minimum 10 MB reads), and bioinformatic analysis (mapping against the reference genome, identification and quantification of transcripts, pairwise comparison of expression levels and determination of significant fold differences) and was conducted by the company. Read statistics and alignment statistics are provided in Table S5. Lowly expressed genes were removed if they had a counts per million value less than 1. Differential gene expression was calculated using the package edgeR (Robinson et al. 2010). Statistical tests were performed for each gene to compare the distributions between conditions generating *p*‐values for each gene. The final *p*‐values were corrected by determining false discovery rates (FDR) using the Benjamin–Hochberg method resulting in an adjusted *p* value. Genes meeting a fold change of > 2 or < 0.5 and an adj_*p*‐value of < 0.001 were considered differentially expressed.

### 4.9 Phylogenetic Tree Inference

For inference of a phylogenetic tree for sHdrR proteins and relatives, proteins were aligned using MAFFT (Katoh and Standley 2013) and the alignment was manually trimmed. A maximum likelihood phylogeny was inferred using IQ‐TREE v1.6.12 (Nguyen et al. 2015). Branch support was calculated by ultrafast bootstrap (2000 replicates) (Hoang et al. 2018). Finally, the tree was displayed using iTol (Letunic and Bork 2021).

### 4.10 Statistics and Reproducibility

Experimental data are expressed as the mean ± standard deviation of the mean (SEM) of the number of tests stated for each experiment. All analyses were reproduced in at least three independent experiments. The significant difference between the two groups was analyzed using an independent student's *t*‐test; the *p*‐value < 0.05 indicated statistical significance.

## Author Contributions

**Jingjing Li:** investigation, writing – original draft, writing – review and editing, visualization. **Nora E. Schmitte:** investigation. **Kaya Törkel:** investigation. **Christiane Dahl:** conceptualization, funding acquisition, supervision, project administration, writing – original draft, writing – review and editing, visualization, validation, data curation.

## Disclosure

The study ensures that all findings are presented truthfully, with proper citations to prior works, and all sources of funding are disclosed.

## Ethics Statement

This study adheres to the ethical standards set by *Molecular Microbiology*. All experimental protocols involving environmental samples, microbial cultures, and data collection were conducted following ethical guidelines and with proper approval from relevant institutional or governmental bodies. The research respects the principles of scientific integrity, transparency, and reproducibility.

## Conflicts of Interest

The authors declare no conflicts of interest.

## Supporting information


Data S1.

Data S2.

Data S3.

## Data Availability

RNA‐Seq raw data files and processed data files are available via the NCBI GEO repository (accession numbers GSE282994 and GSE278992).
