# Supplementary material for: In Hyphomicrobium denitrificans Two Related Sulfane‐Sulfur Responsive Transcriptional Repressors Regulate Thiosulfate Oxidation and Have a Deep Impact on Nitrate Respiration and Anaerobic Biosyntheses
Source: Mol Microbiol. 2025 Jun 26;124(3):204–20. doi: 10.1111/mmi.70002 (PMC12423496; doi:10.1111/mmi.70002)
Supplement: Supplementary file 3 — Data S3. [file MMI-124-204-s002.pdf]

In *Hyphomicrobium denitrificans* two related sulfane-sulfur responsive transcriptional repressors regulate thiosulfate oxidation and have a deep impact on nitrate respiration and anaerobic biosyntheses

Jingjing Li, Nora E. Schmitte, Kaya Törkel, Christiane Dahl*

Corresponding author: ChDahl@uni-bonn.de

Institut für Mikrobiologie & Biotechnologie, Rheinische Friedrich-Wilhelms-Universität Bonn, Bonn, Germany

<u>Supplementary Figures and Tables:</u>

Supplementary Fig. 1: Amino sequence alignment of selected sHdrR homologs

Supplementary Fig. 2: Unrooted phylogenetic tree for sulfane sulfur-responsive ArsR-type transcriptional regulators containing two conserved cysteine residues

Supplementary Fig. 3: Growth of *H. denitrificans* reference and mutant strains on methanol

Supplementary Fig. 4: Growth and thiosulfate consumption of *H. denitrificans* Δ*tsdA* carrying a *shdrR* complementation

Supplementary Fig. 5: Original EMSA gels underlying Figure 3d

Supplementary Fig. 6. Inverted and direct repeats in intergenic regions of the *H. denitrificans* sulfur oxidation locus

Supplementary Fig. 7. Volcano plots of differentially expressed genes for the *H. denitrificans* strains Δ*tsdA*, Δ*tsdA* Δ*shdrR* and Δ*tsdA* Δ*soxR*.

Transcript abundance changes of genes encoding transcriptional regulators and neighboring genes from *Hyphomicrobium denitrificans* Δ*tsdA*

Supplementary Fig. 8. Amino acid sequence alignment of selected LysR-type regulators.

Supplementary Table 1: Occurrence of sHdrR-related proteins with two conserved cysteines (Cys^{50} and Cys^{116} in *Hd*sHdrR)

Supplementary Table 2: mRNAseq analysis of *H. denitrificans* strains *ΔtsdA ΔsoxR* and *ΔtsdA ΔshdrR,* part 1.

Supplementary Table 3: mRNAseq analysis of *H. denitrificans* strains Δ*tsdA* Δ*soxR* and Δ*tsdA* Δ*shdrR,* part 2.

Supplementary Table 4: Strains, primers and plasmids

Supplementary Table 5: RNA-Seq quality control

```
X. fastidiosa BigR         --------------------------------------MVNEMRDDTRPHMTREDMEKRA      22
V. cholera HluY            ------------------------------------------MPYLKGAPMNLQEMEKNS      18
T. mangrovi sHdrR          ---------------------------------------MADGRDKSKVELPLERMQENA      21
H. denitrificans 1NES1     -------------------------------------------------------MRAAA      5
Rp. globiformis            -------------------------------------------------------MENAA      5
Rp. globiformis sHdrR      -------------------------------------------------------MRPNA      5
D. nanyangense             -------------------------------------MRLTEN-VVAPNVVDAGDMLANA      22
Ps. salicylatoxidans SoxR  -------------------------------------MNLPKL-SADQSPEEFEKLLEQA      22
Pc. denitrificans SoxR     MIPAPWIRDKLAQSSGWGCRAPAYPAFRKEGNGDEPLGGIENIRQEPEQEADFDALIKAA      60
H. denitrificans X SoxR    ------------------------------MSGILPNEVIAALEADEEVSPELKRLVLRA      30
Rb. capsulatus SqrR        -------------------------------MGSDTDERC--------AALDAEEMATRA      21
E. coli YgaV               -------------------------------------------------MTELAQLQASA      11
H. denitrificans X sHdrR   ------------------------------MAVVKPRTNRPAVRKARTRQPALHSTDASI      30


X. fastidiosa BigR         NEVANLLKTLSHPVRLMLVCTLVEGEFSV-GELEQQIGIGQPTLSQQLGVLRESGIVETR      81
V. cholera HlyU            AKAVVLLKAMANERRLQILCMLLDNELSV-GELSSRLELSQSALSQHLAWLRRDGLVNTR      77
T. mangrovi sHdrR          TQATGLLKSMANESRLMILCLLSQQEMSV-GELAQRIELSQSALSQHLSILRREKLVKTR      80
H. denitrificans 1NES1     DESSALLKALSNRHRLLVLCQLIDGEKSV-GQLADFLGVRDSTASQHLALLRRDRIIASR      64
Rp. globiformis            EQASELLKSLANRHRLLILCQLVDGERSV-GDLAAFLKTRDSTVSQHLALLRKDGLVQAR      64
Rp. globiformis sHdrR      GRAAGFLKSLANEHRLMILCSLIEGEKSV-GDLQEELNMRQPHLSQQLSRLRLGGLVETR      64
D. nanyangense             RRTTDFLKSLAHEGRLVILCRLSEGPATV-TELEQVLGARQSSVSQQLARLRSEGLVDYE      81
Ps. salicylatoxidans SoxR  RKASDLLKALSHEGRLLILCLLAEGEKSV-SELESIMHMPQAAVSQQLARLRFDRLVNTR      81
Pc. denitrificans SoxR     NDASTFLKALGHDGRLMILCYLMSGPKSV-TELENLLSSRQAVVSQQLARLRHEGLVSAR      119
H. denitrificans X SoxR    RKASDFLKALAHESRLLILCLLAEKERSA-GELENLLSINQPTVSQQLARLRLDGLVQAR      89
Rb. capsulatus SqrR        RAASNLLKALAHEGRLMIMCYLASGEKSV-TELETRLSTRQAAVSQQLARLRLEGLVQSR      80
E. coli YgaV               EQAAALLKAMSHPKRLLILCMLSGSPGTSAGELTRITGLSASATSQHLARMRDEGLIDSQ      71
H. denitrificans X sHdrR   EQATALLRALGSPHRLAILCLLLEGERTV-SEICDKIGARQSLVSQHLTRLRLDGLVKSD      89
                                :*:::.   ** ::* *     :   ::           **:*  :*   ::

X. fastidiosa BigR         RNIKQIFYRLTEAKAAQLVNA---LYTIFCAQEKQA------------------------      114
V. cholera HlyU            KEAQTVFYTLSSTEVKAMIEL---LHRLYCQANQ--------------------------      108
T. mangrovi sHdrR          RESQFVWYSLASEEAERVVHT---LYDIYCADCEID------------------------      113
H. denitrificans 1NES1     RDGQTIWYRIASEPALAVMQV---LNEVYCAGSKSQPGRPRK------------------      103
Rp. globiformis            REAQTIYYSIASCPAQQVLET---LFAIYCSPTPICGMVPAPAESKPPLREPAAAQRTPT      121
Rp. globiformis sHdrR      REAQTIYYSLTDETATEVIGV---LHRRFCKAKPRQRTRQAATRATSAAR------RADP      115
D. nanyangense             RDGRILRYSIADDRARKVVAM---LYDLFCD-----------------------------      109
Ps. salicylatoxidans SoxR  REGRVIYYSIASSEVSSVIST---LYGLFCAPVRKKE-----------------------      115
Pc. denitrificans SoxR     RDGQTIFYSILDPKVVDLLAVLKKLFETDC------------------------------      149
H. denitrificans X SoxR    REGKAVIYSLPDETTRRFIGA---IYDKFCREEPSRKR----------------------      124
Rb. capsulatus SqrR        REGKTIYYSLSDPRAARVVQT---VYEQFCSGD---------------------------      110
E. coli YgaV               RDAQRILYSIKNEAVNAIIAT---LKNVYCP-----------------------------      99
H. denitrificans X sHdrR   RNGYFVSYSLTSAPAQEIIAT---LHKYYCATSAGKRSN---------------------      125
::   : * : .  .  .:     :    *

X. fastidiosa BigR         -------        114
V. cholera HlyU            -------        108
T. mangrovi sHdrR          -------        113
H. denitrificans 1NES1     -------        103
Rp. globiformis            KTPTETK        128
Rp. globiformis sHdrR      ATPDARL        122
D. nanyangense             -------        109
Ps. salicylatoxidans SoxR  -------        115
Pc. denitrificans SoxR     -------        149
H. denitrificans X SoxR    -------        124
Rb. capsulatus SqrR        -------        110
E. coli YgaV               -------        99
H. denitrificans X sHdrR   -------        125
```

Supplementary Fig. 1. Amino sequence alignment of selected sHdrR homologs. Organism names, accession numbers/locus tags and references (if available) in the order of appearance: *Xylella fastidiosa* BigR, XF_0767 (Guimarães et al., 2011), *Vibrio cholera* HlyU, VC_A0642 (Mukherjee et al., 2014; Pis Diez et al., 2023); *Tsuneonella mangrovi*, CJO11_RS12710; *Hyphomicrobium denitrificans* 1NES1, HYPDE_25308 (Venkatramanan et al., 2013); *Rhodopila globiformis*, CCS01_RS26760 and *Rp. globiformis* sHdrR, CCS01_RS13140 (Imhoff et al., 2018); *Devosia nanyangense* HY834_20740 (He et al., 2021), *Pseudaminobacter salicylatoxidans* SoxR, WP_019171658 (Mandal et al., 2007); *Paracoccus denitrificans* SoxR, CAB94376 (Rother et al., 2005), *H. denitrificans* X^T SoxR, Hden_0700 (Li et al., 2023a); *Rhodobacter capsulatus* SqrR, ADE85198 (Shimizu et al., 2017); *Escherichia coli* YgaV, b2667 (Gueuné et al., 2008); *H. denitrificans* X^T sHdrR, Hden_0682 (Li et al., 2023b; Li et al., 2024). An * (asterisk) indicates positions with identical residues. Cysteines are highlighted in yellow. Colons (:) and single dots (.) indicate conserved and semi-conserved amino acids, respectively.

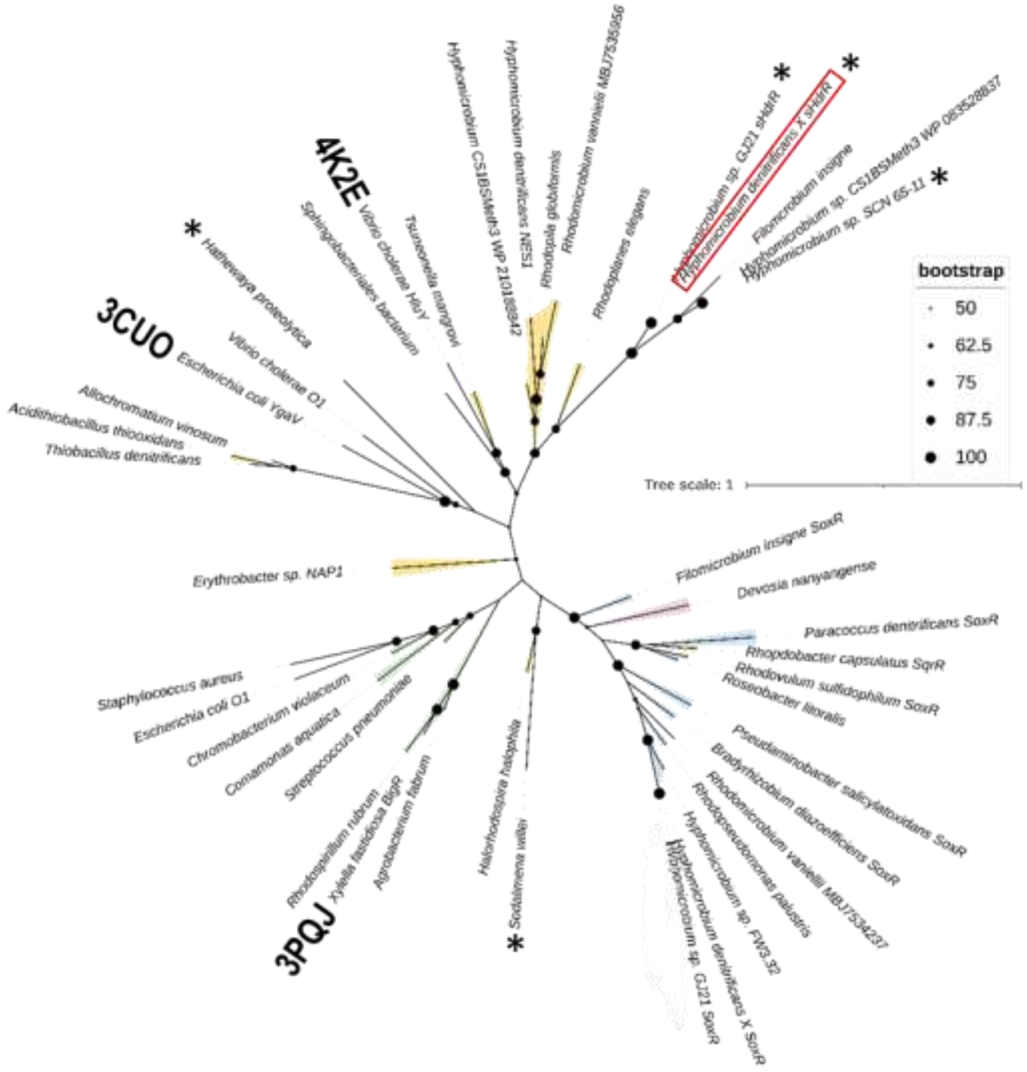

Supplementary Fig. 2. Unrooted phylogenetic tree for sulfane sulfur-responsive ArsR-type transcriptional regulators containing two conserved cysteine residues. Colored ranges indicate the occurrence of the respective gene in immediate vicinity of genes for resistance-nodulation-division (RND) transporters (beige), PmpAB-like YeeE family transporters (green), Sox proteins for thiosulfate oxidation (blue) or sHdr proteins for sulfur oxidation in the cytoplasm (purple). Note that in *Tsuneonella mangrovi* the *shdrR*-like gen is situated next to *shdr* genes and to genes for a RND transporter and that SoxR is encoded between sets of *sox* and RND-encoding genes in *Rhodopseuomonas palustris*. In both cases, only the vicinity to RND-encoding genes is indicated. Asterisks highlight proteins that contain a cysteine at the position equivalent to Cys^{63} sHdrR in the protein from *H. denitrificans* X^{T}. The sHdrR protein from *H. denitrificans* X^{T} is highlighted by a red box. PDB codes are given for structurally characterized proteins. The tree was calculated with 2000 bootstrap resamplings using Ultrafast Bootstrap (Hoang et al., 2018) and IQ-Tree (Trifinopoulos et al., 2016; Minh et al., 2020). Bootstrap values between 50% and 100% are displayed as scaled circles at the branching points. Protein accession numbers, information on adjacent genes and references are available in Supplementary Table 2. The tree is available in Newick format as Supporting information (Supplementary data sHdrR and relatives tree.nwk).

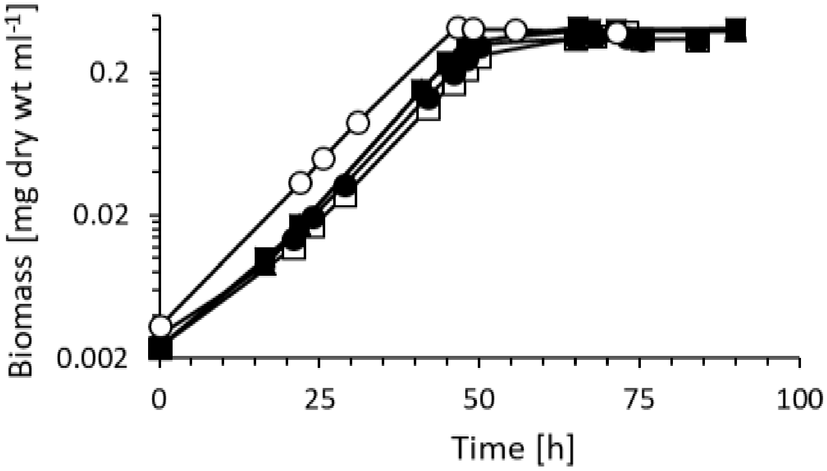

Supplementary Fig. 3. Growth of *H. denitrificans* reference and mutant strains on methanol. a. Growth curves are compared for the reference strain *H. denitrificans* Δ*tsdA* (filled circles), Δ*tsdA* Δ*shdrR* (open boxes), Δ*tsdA* sHdrR $C^{50}S$ (filled boxes), Δ*tsdA* sHdrR $C^{116}S$ (filled triangles), and Δ*tsdA* sHdrR $C^{50}S$ $C^{116}S$ (open circles). Error bars indicating SD for three replicates are too small to be visible for the determination of biomass.

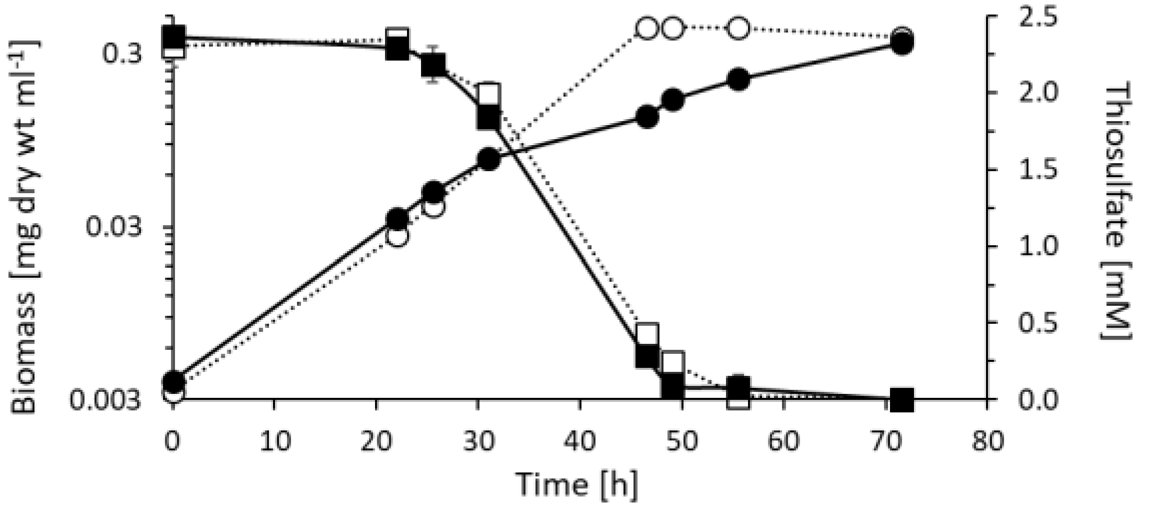

Supplementary Fig. 4. Growth and thiosulfate consumption of *H. denitrificans* ΔtsdA carrying a *shdrR* complementation. Growth curves are shown for medium containing 2 mM thiosulfate. Pre-cultures were either thiosulfate-free (broken lines, open symbols) or were pre-induced and contained 2 mM thiosulfate (solid lines, filled symbols). Values for biomass and thiosulfate are given as circles and boxes, respectively. Error bars indicating SD for three replicates are too small to be visible for the determination of biomass.

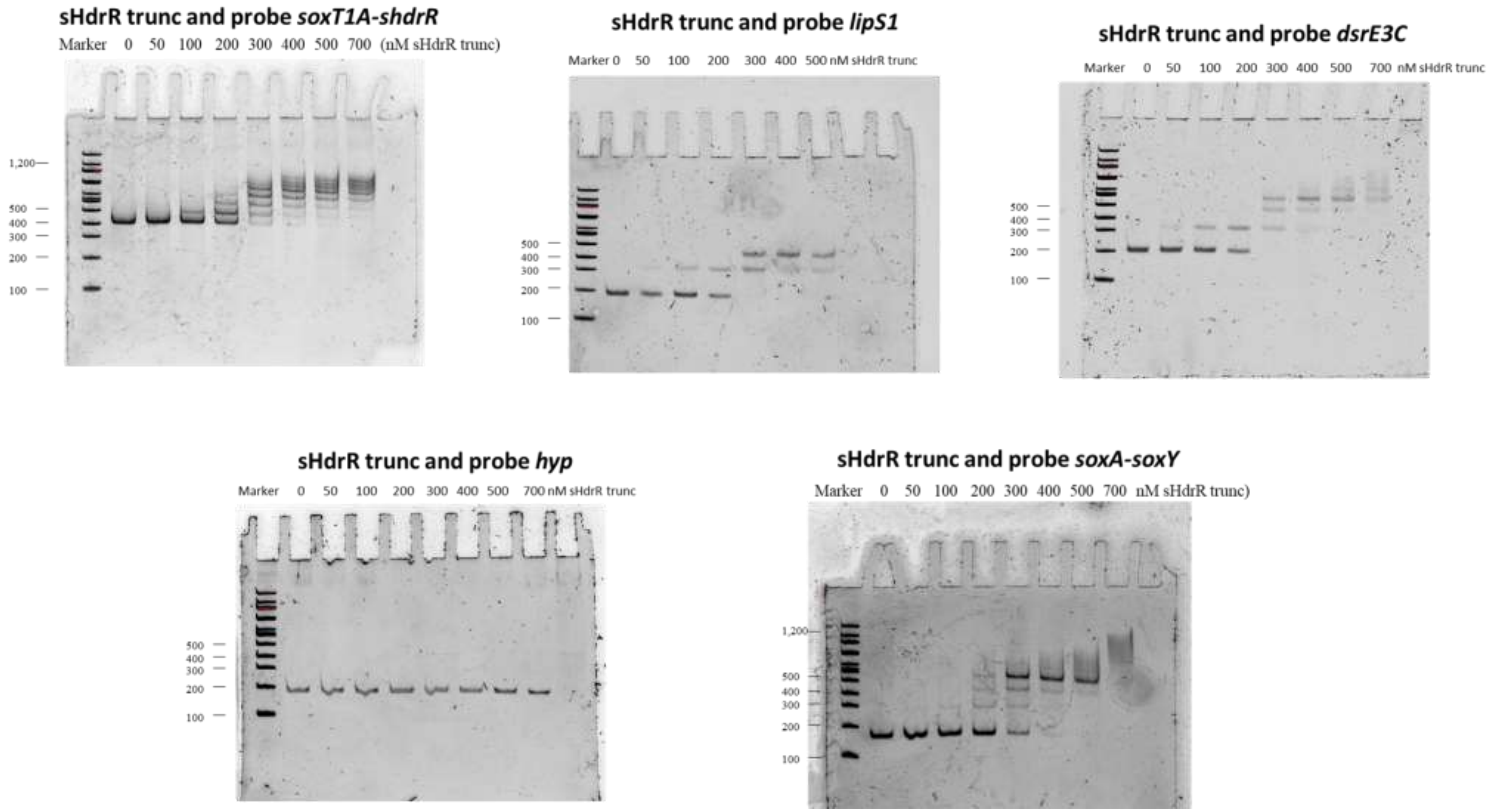

Supplementary Fig. 5. Original EMSA gels underlying Figure 3d.

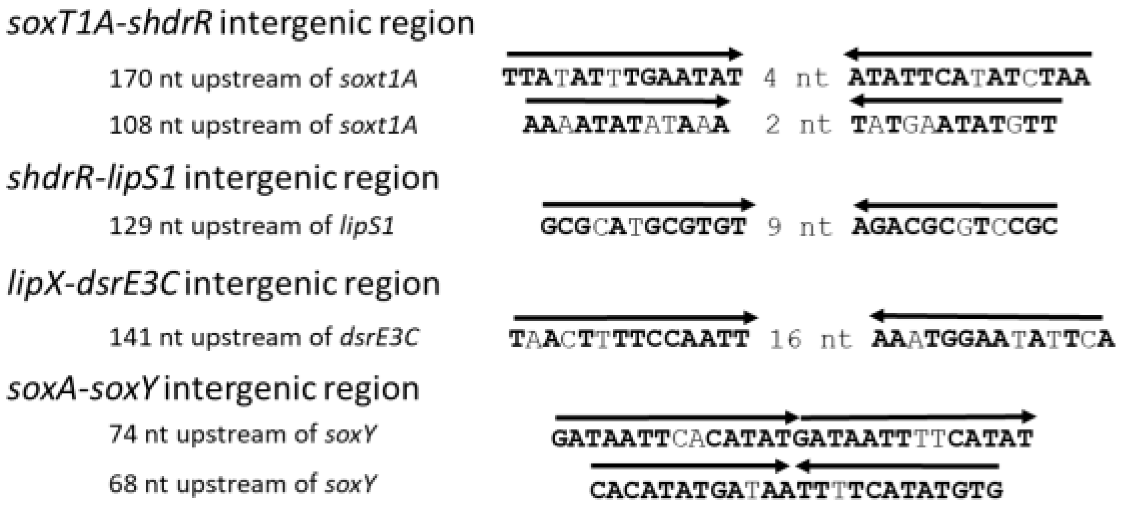

Supplementary Fig. 6. Inverted and direct repeats in intergenic regions of the *H. denitrificans* sulfur oxidation locus. Matching nucleotides are printed in bold.

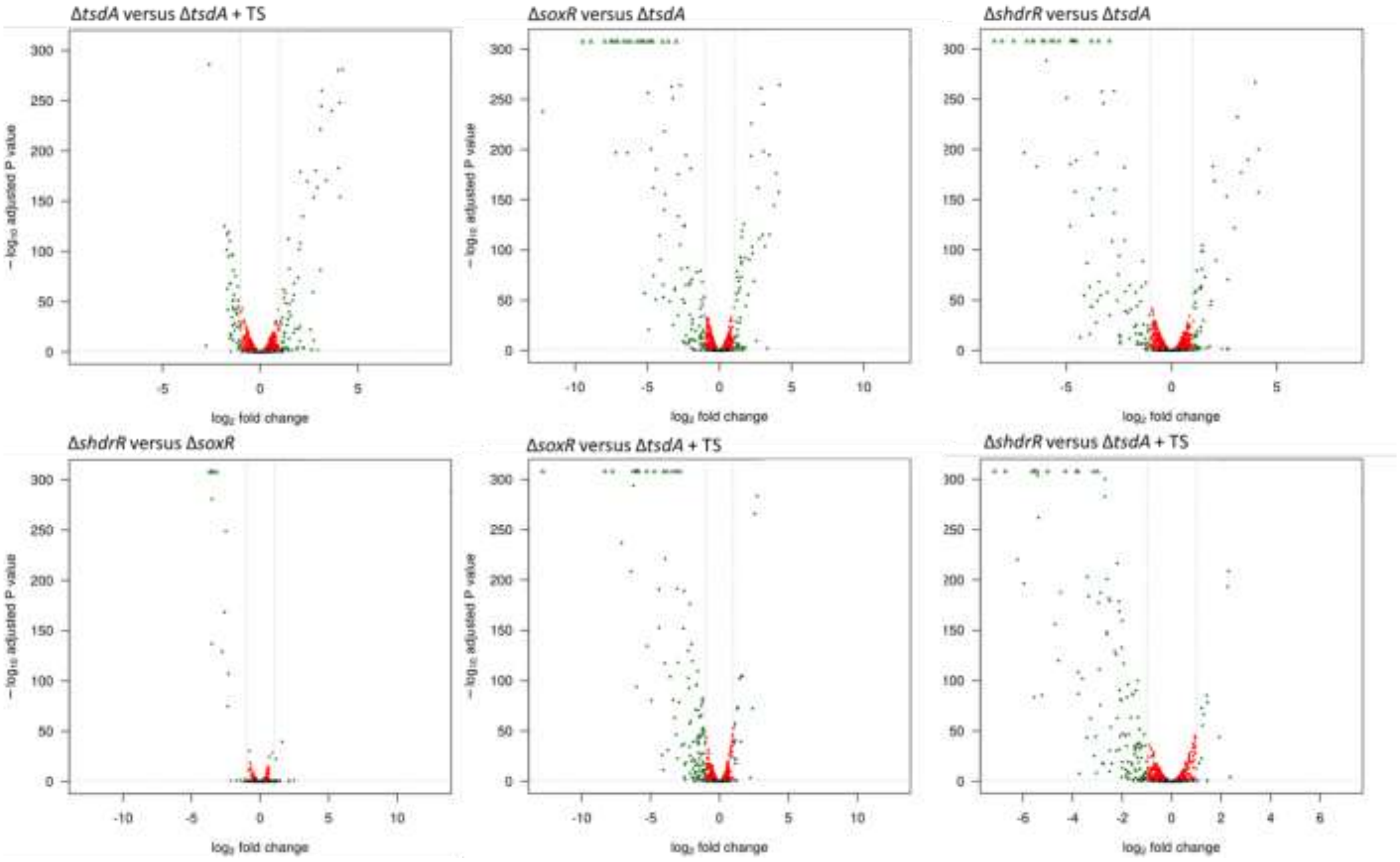

Supplementary Fig. 7. Volcano plots of differentially expressed genes for the *H. denitrificans* strains Δ*tsdA*, Δ*tsdA* Δ*shdrR* and Δ*tsdA* Δ*soxR*. The strains/conditions compared are given on top of each panel. TS, thiosulfate. • significant and has >2-fold change, • significant (FDR corrected *p*-value <= 0.1), • not significant.

```
CcmR            MGHHHHHHMQATLHQLKVFEATARHGSFTRAAEELYITQPTVSSQIKQLSKTVGLPLFEQ 60
YeiE            -----------TLRQLEVFAEVLKSGSTTQASQMLSLSQSAVSAALTDLEGQLGVQLFDR 49
Hden_0835       ----------MTLEQLRIFVAVAEREHVTQAAKELNLTQSATSAAVSALEARYATKLFDR 50
                           **.**.:*  . .    *:*:: * ::* :.*: :. *.   .  **::

CcmR            IGKRLYLTEAGQELLVTCQDIFQRLDNFAMKVADIKGTKQGRLRLAVI-TTAKYFIPRLL 119
YeiE            VGKRLVVNEHGRLLYPRTVALLEQAGEIERL----FRNDNGAIRVYASSTIGNYILPEII 105
Hden_0835       IGRRIVLTQAGKLFLVEAKSVLARAAAAEKVLADLAGLERGSLRIGASQTAGNYWLPEII 110
                :*:*: :.: *: :      :: :              ..* :*: .  * .:* :*.::

CcmR            GEFIQKYPGIEVSLKVTNHEQIRHRMQNNEDDLYIVSEPPEEIDLNYQPFLDNPLVVIAR 179
YeiE            ARYRRDFPDLPLEMSVGNSLDVVQAVCDFRVDIGLIEGPCHMAEIVAQPWLEDELVVFAS 165
Hden_0835       HRYQSLFPGISIALKIGNTETVAADVEDGVADLGFIEGEIDNPVLSVTPVADDDMVLVVA 170
                 .:   :*.: : :.: *   :   : :   *: ::.   .   :   *  :: :*:..

CcmR            RDHPLAGKSNIPITALNDEAFIMREKGSGTRLAVQNLFHR---HYVDVRVRLELGSNEAI 236
YeiE            PASPLLEGEV-TLERLAAMPWILREKGSGTREIVDYLLLS---HLPQFRLSMELGNSEAI 221
Hden_0835       PNNPLAKQPLRALSQIAQARWVVREAGSGTRAILEADVAKLGIDPKSLDIALELPSNEAV 230
                   **       :  :    :::** *****  ::  .     .  .. : :** ..**:

CcmR            KQAIAGGMGISVLSQHTLVSEGARSELTILDIDEFPIKRRWYVANLAGKQLSVITQTFLD 296
YeiE            KHAVRHGLGVSCLSRRVIAEQLETGSLVEVKVPLPPLVRTLYRIHHRQKHLSSALARFLR 281
Hden_0835       RGAVVAGSGITILSRLVVAAPLKAKTLVALDVPLPAR--KFFALRHKERYFTRAERTFID 288
                : *:  * *:: **: .:.       *. :.:         :  .   : ::     *:

CcmR            YLMAVTKNMPAPFAEQLTTQQTPVKLVL        324
YeiE            YCEL------------------------        285
Hden_0835       VATGKQSSRAPG----------------        300
```

Supplementary Fig. 8. Amino acid sequence alignment of selected LysR-type regulators. The protein deduced from Hden_0635 is aligned with CcmR/NdhR from *Synechocystis* PCC6803 (PDB 5Y2V) and YeiE from *Cronobacter sakazakii* (GenBank accession number ELY4740156). For *Synechocystis* CcmR/NdhR the residues interacting with 2-phosphoglycolate, which is an inducer (Jiang et al., 2018), are highlighted in green. For YeiE the residues interacting with sulfite (Hong et al., 2022) are marked yellow. An * (asterisk) indicates positions with identical residues. Cysteines are highlighted in yellow. Colons (:) and single dots (.) indicate conserved and semi-conserved amino acids, respectively.

Supplementary Table 1. Occurrence of sHdrR-related proteins with two conserved cysteines (Cys^{50} and Cys^{116} in *Hds*HdrR). Accession number and/or locus tags are provided. Linked genes were manually analyzed. Furthermore, genomes were checked via HMSS2 (Tanabe and Dahl, 2023) for the presence of genes encoding Sox-dependent thiosulfate oxidation in the periplasm (set positive when *soxYZAXB* were detected, thus covering complete and truncated Sox systems (Li et al., 2023a)) and sHdr-driven sulfane sulfur oxidation in the cytoplasm (set positive when at least 70 % of the genes *shdrC1B1AHC2B2* or *shdrC1B1AHB3etfAB* were present in a syntenic block, respectively (Kümpel et al., 2024)).

Organism	Accession, locus tag	Linked genes	sHdr system	Sox system	References
**Pseudomonadota**					
**Alphaproteobacteria**					
**Hyphomicrobiales**					
Hyphomicrobiaceae					
Hyphomicrobium denitrificans X^T (ATCC 51888^T)	sHdrR: Hden_0682<br>SoxR: Hden_0700	*shdr-lbpA*<br>*sox*	Yes	Yes	
Hyphomicrobium denitrificans 1NES1	HYPDE_25308	RND transporter	No	No	(Venkatramanan et al., 2013)
Hyphomicrobium sp. GJ21	sHdrR: HYPGJ_30422<br>SoxR: HYPGJ_30404	*shdr-lbpA*<br>*sox*	Yes	Yes	(Tatusova et al., 2014)
Hyphomicrobium sp. SCN 65-11	ABS54_17655	Short fragment	No	No	(Kantor et al., 2015)
Hyphomicrobium sp. CS1BSMeth3	WP_083528837: CS1BSM3_04686<br>WP_210188842: CS1BSM3_RS16485	TauE, *ccm* genes<br>RND transporter	Yes	Yes	Adelskov and Patel, unpublished
Hyphomicrobium sp. FW.3.32	CTY20_06775	*soxBZYAX*	No	Yes	(Zhang et al., 2017)
Filomicrobium insigne CGMCC 1.6497^T	SAMN04488061_2704<br>SoxR: SAMN04488061_1979	Only *soxYZ*<br>*soxCBZY*	No	Yes	(Wu et al., 2009)
Rhodomicrobium vaniielii ATCC 17100^T	MBJ7534237 JDN40_08990<br>MBJ7535956 JDN40_17755	*tauE*<br>RND transporter	No	SoxXA present	Conners et al, unpublished
Devosiaceae					
Devosia nanyangense NC_groundwater_1586_Pr3_B-0.1um_66_15	HY834_20740	*shdr* gene cluster, *tusA*	Yes	No	(He et al., 2021)
**Acetobacterales**					
Acetobacteriaceae					
Rhodopila globiformis DSM 161^T	CCS01_RS26760<br>CCS01_RS13140	RND transporter, Rhd<br>*shdr* genes	Yes	No	(Imhoff et al., 2018)
**Rhizobiales**					
Rhizobiaceae					
Agrobacterium fabrum (tumefaciens) C58^T	BIGR_AGRFC, Atu3466	Rhd-PDO fusion-*bigR-pmpBA*	No	No	(Goodner et al., 2001; Guimarães et al., 2011)
Pseudaminobacter salicylatoxidans KCT001	WP_019171658	*sox*	No	Yes	(Mandal et al., 2007)

Xanthobacteraceae					
Bradyrhizobium diazoefficiens USDA10^T	BAC48771	*sox*	No	Yes	(Kaneko et al., 2002)
Rhodopseudomonas palustris TIE-1	Rpal_4967	Between *sox* genes and genes for RND transporter	No	Yes	(Larimer et al., 2004)
Rhodoplanes elegans DSM 11907^T	RAI38494, CH338_12525	RND transporter, sulfurtransferase	No	Yes	(LaSarre et al., 2018)
**Rhodospirillales**					
Rhodospirillaceae					
Rhodospirillum rubrum ATCC 11170^T	WP_011389407, ABC22517, Rru_A1717	*pmpB-like*	No	No	(Munk et al., 2011)
**Rhodobacterales**					
Paracoccaceae					
Paracoccus denitrificans GB17	CAB94376	*sox* genes	No	Yes	(Wodara et al., 1997; Rother et al., 2005)
Rhodobacteraceae					
Roseobacter litoralis Och 149^T	AEI95148	*sox* genes	No	Yes	(Kalhoefer et al., 2011)
Rhodobacter capsulatus SB 1003	ADE85198	RND transporter	No	No	(Shimizu et al., 2017; Capdevila et al., 2021)
Rhodovulum sulfidophilum DSM 1374^T	AAO11780	*sox* genes	No	Yes	(Appia-Ayme et al., 2001)
**Sphingomonadales**					
Sphingomonadaceae					
Tsuneonella (Altererythrobacter) mangrovi CD9-11^T	WP_240504499: CJO11_RS12710	close to *shdr* genes and genes for RND transporter	Yes	No	(Tatusova et al., 2014)
Erythrobacter sp. NAP1	EAQ29854, NAP1_03740	RND efflux system	No		(Koblizek et al., 2011)
**Gammaproteobacteria**					
**Chromatiales**					
Chromatiaceae					
Allochromatium vinosum DSM 180^T	Alvin_3027	Rhd	No	Yes	(Weissgerber et al., 2011)
**Nitrococcales**					
Ectothiorhodospiraceae					
Halorhodospira halophila DSM244^T	Hhal_1425	RND transporter	No	Yes	(Challacombe et al., 2013)
**Enterobacterales**					
Enterobacteriaceae					
Escherichia coli O1 strain PSU-0611	EEZ6061186, DCO30_005030	Short fragment	No	No	(Lacher et al., 2020)
Escherichia coli K12 substr. MG1655	b2667; YgaV PDB: 3CUO	YgaP: membrane-associated protein with rhodanese activity	No	No	(Paul and Larson, 2006; Riley et al., 2006; Gueuné et al., 2008)

Vibrionaceae					
Vibrio cholera O1 biovar El Tor N16961	HlyU, VC_0678, HLYU_VIBCH, PDB: 4K2E<br>VC_A0642, AAF96543	Transcriptional activator of hemolysin,	No	No	(Williams et al., 1993; Heidelberg et al., 2000; Mukherjee et al., 2014)
**Xanthomonadales**					
Xanthomonadaceae					
Xylella fastidiosa 9a5c	WP_010893290, XF_0767, PDB: 3PQJ	Blh: DUF442-PDO fusion (XF_0768) *pmpBA* (XF_0765, 0766)	No	No	(Simpson et al., 2000; Barbosa and Benedetti, 2007; Guimarães et al., 2011)
**Burkholderiales**					
Chromobacteriaceae					
Chromobacterium violaceum ATCC 12472[T]	CV_0084	*pmpAB,* Cyt c4	No	No	(Brazilian National Genome Project, 2003)
Burkholderiaceae					
Comamonas aquatica CJG	WP_045267543	*pmpAB,* DUF599 family	No	No	(Dai et al., 2016)
Thiobacillaceae					
Thiobacillus denitrificans ATCC 25No250	AAZ98348, Tbd_2395	alone	No	Yes	(Beller et al., 2006)
**Acidithioacillales**					
Acidithiobacillaceae					
Acidithiobacillus thiooxidans ATCC 19377[T]	WP_024893036.1, GCD22_RS14465	RND transporter	Yes	Yes	(Valdes et al., 2011)
**Bacteroidota**					
**Bacteroidia**					
**Chitinophagales**					
Sphingobacteriales bacterium PMG_127	RYD90138	PmpA or B (short contig)	-	-	(Crombie et al., 2018)
**Bacillota**					
**Bacilli**					
**Lactobacillales**					
Streptpcoccaceae					
Streptococcus pneumonia SMRU2535	CJK49847, ERS022045_00348	*pmpBA*, uncharacterized, Rhd, sulfate permease, sulfide dehydrogenase	No	No	(Chewapreecha et al., 2014)
**Staphylococcales**					
Staphylococcaceae					
Staphylococcus aureus VB1919	RTY94661	Short contig	No	No	Balaji and Yamuna unpublished

**Clostridia**					
**Clostridiales**					
Clostridiaceae					
Hathewaya proteolytica DSM 3090^T	SHJ53940, SAMN02745248_00335	Upstream two genes for FeS containing proteins, then two for sulfur carrier protein ThiS	No	No	Joint Genome Institute
**Cyanobacteriota**					
**Cyanobacteriia**					
**Cyanobacteriales**					
Geitlerinemaceae					
Sodalimena (former *Phormidium*) *willei* BDU 130791	OAB56254, AY600_15135	All hypothetical	No	No	Peter. et al unpublished

Ccm, cytochrome c maturation (Thöny-Meyer, 2002); PDO, persulfide dioxygenase; PmpAB, members of the YeeE/YedE family of transporters that have been predicted to transport sulfur-containing ions (Gristwood et al., 2011); Rhd, rhodanese; RND, Resistance-nodulation-division family transporters, a category of bacterial efflux pumps, especially identified in Gram-negative bacteria (Nikaido, 2011); TauE, sulfite exporter (Weinitschke et al., 2007)

Supplementary Table 2. mRNAseq analysis of *H. denitrificans* strains *ΔtsdA ΔsoxR* and *ΔtsdA ΔshdrR*, part 1. Genes with higher mRNA abundance in the regulator-deficient mutants than in the reference strain in the absence of thiosulfate.

Locus tag	Annotation[a]	***ΔtsdA ΔsoxR* vs *ΔtsdA*** Fold change**	***ΔtsdA ΔshdrR* vs *ΔtsdA*** Fold change**
**Sulfur metabolism**			
Hden_0678	hypothetical protein	5.82	3.51
Hden_0679	DsbA family protein	11.06	2.17
Hden_0680	Rhd, sulfur transferase domain-containing protein	15.61	2.60
Hden_0681	SoxT1A, YeeE/YedE family protein	24.43	4.32
Hden_0683	LipS1, radical SAM protein	17.88	25.64
Hden_0684	LipT, NAD(P)/FAD-dependent oxidoreductase	17.27	17.42
Hden_0685	LipS2, radical SAM protein	14.96	15.70
Hden_0686	Lpl(AB), lipoate--protein ligase family protein	16.04	18.56
Hden_0687	LipX, GMP synthase - glutamine amidotransferase domain-like protein	15.25	17.49
Hden_0688	DsrE3C, DsrE/DsrF/DrsH-like family protein	10.84	15.72
Hden_0689	sHdrC1	8.19	11.79
Hden_0690	sHdrB1	7.40	10.69
Hden_0691	sHdrA, FAD-dependent oxidoreductase	8.21	11.65
Hden_0692	sHdrH	7.93	12.36
Hden_0693	sHdrC2	6.72	9.96
Hden_0694	sHdrB2	6.37	8.71
Hden_0695	sHdrI	5.24	7.90
Hden_0696	LbpA2	4.17	6.12
Hden_0697	Cytochrome P450	4.77	ns
Hden_0698	TusA family sulfurtransferase	4.88	ns
Hden_0699	SoxT1B, YeeE/YedE family protein	2.69	ns
Hden_0701	SoxS, thioredoxin family protein	13.69	ns
Hden_0702	Sulfur oxidation c-type cytochrome SoxX	12.43	ns
Hden_0703	Sulfur oxidation c-type cytochrome SoxA	17.19	ns
Hden_0704	Thiosulfate oxidation carrier protein SoxY	17.44	ns
Hden_0705	Thiosulfate oxidation carrier complex protein SoxZ	16.72	ns
Hden_0706	Thiosulfohydrolase SoxB	14.50	ns
Hden_0834	YeiH family protein, sulfite export	ns	3.70
**Carbon metabolism**			
Hden_0802	DUF3734 domain-containing protein	2.42	ns
Hden_2747	Acyl CoA:acetate/3-ketoacid CoA transferase	2.33	2.12
**Heme degradation and iron acquisition**			
Hden_0540	TonB-dependent heme receptor	4.61	ns
Hden_0541	Heme degrading monooxygenase HmoA	3.45	3.02
Hden_0542	TonB family protein	3.20	2.82
Hden_0874	Hemin uptake protein HemP	3.53	3.58
Hden_0875	Heme degrading monooxygenase HmoA	2.86	2.62
Hden_0876	Heme utilization cystosolic carrier protein ChuX/HutX	2.88	2.69
Hden_0877	Heme transport system substrate-binding protein ChuT	2.97	2.77

Hden_0878	Heme transport system permease protein ChuU	2.82	2.74
Hden_0879	Heme transport system ATP-binding protein, HmuV	2.64	2.68
Hden_1331	TonB-dependent siderophore receptor	2.54	2.24
Hden_1332	PepSY domain-containing protein	2.23	ns
Hden_1333	hypothetical protein	2.25	ns
Hden_3200	hypothetical protein	10.51	8.75
Hden_3201	hypothetical protein	9.99	7.27
Hden_3202	Hemin uptake protein HemP	8.85	6.35
**Transport**			
Hden_0532	ABC transporter substrate-binding protein, branched chain amino acid transport	2.73	ns
Hden_2931	Potassium-transporting ATPase subunit KdpA	2.92	2.08
Hden_3198	YceI family protein, periplasmic, polyisoprenoid-binding	2.23	2.04
Hden_3199	Cytochrome *b*, YceJ	2.64	2.31
**Regulation**			
Hden_0594	helix-turn-helix domain-containing protein	2.60	2.39
Hden_0722	response regulator transcription factor, LuxR family	2.58	
Hden_2164	AraC family transcriptional regulator	3.05	ns
**Respiration and electron transport**			
Hden_2084	pseudoazurin	4.56	4.11
Hden_3539	cupredoxin domain-containing protein	2.40	2.12
Hden_2748	c-type cytochrome	3.50	3.17
Hden_2908	cytochrome c oxidase subunit II	2.32	2.33
**Other**			
Hden_0136	hypothetical protein	1.00	2.27
Hden_0441	glycosyltransferase	1.00	6.50
Hden_0457	hypothetical protein	2.29	2.30
Hden_0523	zf-HC2 domain-containing protein	ns	2.25
Hden_0525	catalase family peroxidase	ns	3.92
Hden_0738	hypothetical protein	2.18	2.23
Hden_0914	hypothetical protein	ns	5.23
Hden_0990	hypothetical protein	ns	2.01
Hden_1114	hypothetical protein	2.51	2.34
Hden_1235	phage GP46 family protein	ns	2.06
Hden_1416	hypothetical protein	2.01	ns
Hden_1432	DUF3307 domain-containing protein	ns	2.26
Hden_1518	hypothetical protein	ns	2.28
Hden_2458	hypothetical protein	9.73	6.35
Hden_2517	hypothetical protein	2.49	ns
Hden_2518	catalase	2.85	2.73
Hden_2542	class I SAM-dependent methyltransferase	ns	2.15
Hden_2599	hypothetical protein	2.31	2.54
Hden_2684	hypothetical protein	ns	2.30
Hden_2944	DUF3302 domain-containing protein	2.01	ns
Hden_2965	hypothetical protein	ns	2.41
Hden_2982	hypothetical protein	ns	2.35
Hden_3015	DNA cytosine methyltransferase	ns	2.22
Hden_3020	hypothetical protein	ns	2.70
Hden_3022	hypothetical protein	3.27	ns
Hden_3142	FHA domain-containing protein	2.14	2.10
Hden_3444	hypothetical protein	ns	2.19

Hden_3518	hypothetical protein	2.54	2.18
Hden_R0029		2.00	2.21
Hden_R0052		ns	2.47

ns, not significant

[a] Gene names obtained using sequence similarities in Uniprot or NCBI databases

** Significance threshold set at >2-fold change and FDR-corrected $p<0.001$;

Supplementary Table 3. mRNAseq analysis of *H. denitrificans* strains *ΔtsdA ΔsoxR* and *ΔtsdA ΔshdrR*, part 2. Genes with lower mRNA abundance in the regulator-deficient mutants than in the reference strain in the absence of thiosulfate. Potential regulatory proteins associated with genes for respiratory proteins are printed in bold and not arranged under the headline "regulation".

Locus tag	Annotation[a]	***ΔtsdA ΔsoxR* vs. *ΔtsdA*** Fold change**	***ΔtsdA ΔshdrR* vs. *ΔtsdA*** Fold change**
**Biosynthesis of metabolites and cofactors**			
**PQQ**			
Hden_0547	FmdE family protein, Flag1 repressor motif	0.175	0.184
Hden_0550	urate hydroxylase PuuD	ns	0.427
Hden_0551	pyrroloquinoline quinone biosynthesis protein PqqE	0.424	0.362
Hden_0552	pyrroloquinoline quinone biosynthesis peptide chaperone PqqD	0.190	0.185
Hden_0553	pyrroloquinoline quinone precursor peptide PqqA	0.232	0.321
**Fatty acids**			
Hden_0554	beta-ketoacyl-ACP synthase FabF	0.067	0.056
Hden_0555	beta-ketoacyl-ACP synthase FabF	0.057	0.036
Hden_0556	zinc-binding dehydrogenase, putative enoyl-ACP reductase FabI function	0.012	0.008
Hden_0557	beta-ketoacyl-ACP synthase FabF	0.007	0.012
Hden_0558	beta-ketoacyl-ACP synthase FabF	0.010	0.016
Hden_0559	beta-hydroxyacyl-ACP dehydratase FabZ	0.004	0.006
Hden_0560	acyl carrier protein	0.001	0.003
Hden_0561	3-oxoacyl-ACP reductase, FabG	0.002	0.004
Hden_0562	HAD-IIIC family phosphatase, putative involvement in methoxymalonyl-ACP biosynthesis, FkbH-like protein	0.025	0.038
Hden_0563	acyl carrier protein	0.199	0.218
**Ubiquinone**			
Hden_0564	UbiX family flavin prenyltransferase	0.219	0.049
Hden_0565	UbiD family decarboxylase	0.033	0.068
Hden_0566	UbiT ubiquinone biosynthesis accessory factor UbiT, SCP2 sterol-binding domain-containing protein	0.046	0.071
Hden_0567	O_2-independent ubiquinone biosynthesis protein UbiU	0.028	0.073
Hden_0568	O_2-independent ubiquinone biosynthesis protein UbiV	0.107	0.084
Hden_0569	Cytochrome P450	0.185	0.168
**Hden_0570**	**Crp/Fnr family transcriptional regulator**	0.138	0.093
Hden_0571	DUF2478 domain-containing protein	0.411	0.421
**Respiration and electron transport**			
Hden_0508	Cupin domain-containing protein	0.173	0.238
Hden_0509	SPW repeat protein	0.238	0.321
Hden_0510	Ferredoxin-NADP reductase	0.200	0.211
Hden_0572	Hypothetical protein	0.042	0.097
Hden_0573	4Fe-4S binding protein	0.047	0.036
Hden_0574	Periplasmic cupredoxin domain-containing protein	0.032	0.032
Hden_0579	NorE	ns	0.423
Hden_0581	Nitric oxide reductase subunit C, NorC	0.066	0.068
Hden_0582	Nitric oxide reductase subunit B, NorB	0.215	0.177
Hden_0583	Nitric oxide reductase NorQ protein	0.453	0.396

Hden_0584	Nitric oxide reductase NorD protein, VWA domain-containing protein	0.457	0.486
Hden_0585	Cytochrome c, hypothetical protein	0.388	0.461
Hden_0587	DUF2946 domain-containing protein	0.336	0.376
Hden_0589	Hypothetical protein	0.137	0.131
Hden_0590	NnrS family protein, involved in response/tolerance to NO	0.309	0.251
Hden_0591	Copper-containing nitrite reductase apoprotein NirK	0.189	0.213
Hden_0592	Host attachment family protein	0.042	0.042
**Hden_0595**	**Helix-turn-helix domain-containing protein**	0.037	0.043
**Hden_0596**	**PAS domain-containing protein**	0.259	0.309
**Hden_0597**	**Signal transduction histidine kinase, nitrite/nitrate specific NarQ**	0.073	0.074
**Hden_0598**	**Two-component system response regulator NarL**	0.059	0.062
Hden_0673	NitT/TauT family transport system substrate-binding protein, nitrate/sulfonate transport	0.138	0.154
Hden_0674	NitT/TauT family transport system permease protein	0.243	0.217
Hden_0675	NitT/TauT family transport system ATP-binding protein	0.271	0.297
Hden_0676	NnrS family protein, involved in response/tolerance to NO	0.317	0.349
Hden_0677	NnrS family protein, involved in response/tolerance to NO	0.491	0.412
Hden_0922	VOC family protein	0.201	0.326
Hden_0924	Porin	0.086	0.090
Hden_0925	NarK, nitrate/nitrite antiporter	0.019	0.015
Hden_0926	Nitrate reductase subunit alpha, NarG	0.179	0.152
Hden_1054	Cytochrome c	0.160	0.172
Hden_1055	Cytochrome c_{550} domain protein	0.007	0.010
Hden_1483	Cytochrome c family protein	0.480	1.000
Hden_1879	Permease protein NosY, copper transport	0.408	1.000
Hden_1880	ABC transporter ATP-binding protein NosF, copper transport	0.466	0.460
Hden_1881	Periplasmic nitrous oxide reductase family maturation protein NosD	0.266	0.306
Hden_1882	TAT-dependent nitrous-oxide reductase NosZ	0.126	0.130
Hden_1883	NosR/NirI family protein	0.031	0.037
Hden_1884	Ferritin family protein	0.247	0.253
Hden_1937	NADH-quinone oxidoreductase subunit NuoF	0.481	1.000
Hden_2045	FixH family protein	0.107	0.107
Hden_2046	Cytochrome c oxidase accessory protein CcoG	0.064	0.071
Hden_2047	Cytochrome-*c* oxidase cbb_3-type subunit III	0.012	0.014
Hden_2048	cbb3-type cytochrome c oxidase subunit 3	0.006	0.010
Hden_2049	Cytochrome-*c* oxidase cbb_3-type subunit II	0.007	0.011
Hden_2050	Cytochrome-*c* oxidase cbb_3-type subunit I	0.005	0.008
**Carbon metabolism**			
Hden_0042	Poly(3-hydroxybutyrate) depolymerase	0.435	0.484
Hden_0607	NAD-dependent formate dehydrogenase	3.915	2.864
**Sulfur metabolism**			
Hden_0759	SufS family cysteine desulfurase	ns	0.472
Hden_1046	Sulfate adenylyltransferase subunit CysN	0.250	0.388
Hden_1047	Sulfate adenylyltransferase subunit CysD	0.426	ns

Hden_1491	NADPH-dependent assimilatory sulfite reductase hemoprotein subunit, CysI	0.433	ns
**Transport**			
Hden_2042	Sulfite exporter TauE/SafE family protein	0.438	0.376
Hden_2044	cadmium-translocating P-type ATPase	0.151	0.142
Hden_2136	DHA2 family efflux MFS transporter permease subunit	0.384	0.261
**Heme degradation and iron acquisition**			
Hden_0575	FtrA, periplasmic iron binding protein	0.014	0.019
Hden_0576	HemN, oxygen-independent coproporphyrinogen III oxidase	0.040	0.043
Hden_0599	Heme anaerobic degradation, anaerobilin synthase ChuW/HutW	0.239	0.185
**Regulation**			
Hden_0099	PAS domain-containing protein	0.118	0.120
Hden_2177	Crp/Fnr family transcriptional regulator	0.022	0.021
Hden_2274	NnrS family protein, involved in response to NO	0.391	0.360
Hden_3436	response regulator	0.441	1.000
**Other**			
Hden_0086	Group II truncated hemoglobin	0.092	0.087
Hden_0095	HPF/RaiA family ribosome-associated protein	0.070	0.085
Hden_0096	Zinc-dependent alcohol dehydrogenase family protein	0.036	0.040
Hden_0097	Flavin reductase family protein	0.070	0.074
Hden_0174	Hypothetical protein	0.261	1.000
Hden_0328	Hypothetical protein	0.399	0.361
Hden_0672	TonB-dependent receptor	0.150	0.151
Hden_0959	5-aminolevulinate synthase	0.400	0.430
Hden_1119	Phage tail tape measure protein	ns	0.444
Hden_1171	Hypothetical protein	0.491	ns
Hden_1773	radical SAM protein	0.272	0.313
Hden_1841	Universal stress protein	0.099	0.102
Hden_1876	Hypothetical protein	0.038	0.043
Hden_2272	Membrane protein	0.276	0.180
Hden_2281	HD domain-containing protein	ns	0.464
Hden_2596	Alpha/beta fold hydrolase	0.395	0.367
Hden_2615	Hypothetical protein	0.117	0.092
Hden_2827	Ferric reductase-like transmembrane domain-containing protein	0.024	0.025
Hden_2910	Hypothetical protein	0.397	0.458
Hden_3135	Circularly permuted type 2 ATP-grasp protein	0.487	ns

ns, not significant
[a] Gene names obtained using sequence similarities in Uniprot or NCBI databases
** Significance threshold set at <0.5-fold change and FDR-corrected *p*<0.001;

Supplementary Table 4. Strains, primers and plasmids

Strains primers or plasmids	Relevant genotype, description or sequence	Reference or source
**Strains**		
Escherichia coli 10-beta	*Δ(ara-leu) 7697 araD139 fhuA ΔlacX74 galK16 galE15 e14- Φ80dlacZΔM15 recA1 relA1 endA1 nupG rpsL* (Str^{R}) *rph spoT1 Δ(mrr-hsdRMS-mcrBC)*	New England Biolabs
E. coli DH5α	F^{-} Φ80*lac*ZΔM15 Δ(*lac*ZYA-*arg*F)U169 *rec*A1 *end*A1 *hsd*R17(r$_{K}^{-}$, m$_{K}^{+}$) *pho*A *sup*E44 λ^{-}*thi*-1 *gyr*A96 *rel*A1	New England Biolabs
E. coli BL21(DE3)	F^{-}*omp*T *hsd*S$_{B}$ (r$_{B}^{-}$, m$_{B}^{-}$) *gal dcm* (DE3)	Novagen
Hyphomicrobium denitrificans Δ*tsdA*	Sm^{R}, in-frame deletion of *tsdA* in *H. denitrificans* Sm200	(Koch and Dahl, 2018)
H. denitrificans Δ*tsdA* Δ*shdrR*	Sm^{R}, in-frame deletion of *shdrR* (Hden_0682) in *H. denitrificans* Δ*tsdA*	(Li et al., 2023b)
H. denitrificans Δ*tsdA* Δ*soxR*	Sm^{R}, deletion of *soxR* (Hden_0700) in *H. denitrificans* Δ*tsdA*	(Li et al., 2023a)
H. denitrificans Δ*tsdA shdrRcomp*	Sm^{R}, *cis* complementation of *H. denitrificans* Δ*tsdA* Δ*shdrR* with *shdrR*	This work
H. denitrificans Δ*tsdA shdrR-Cys^{50}Ser*	Exchange of sHdrR-Cys^{50} to Ser in *H. denitrificans* Δ*tsdA*	This work
H. denitrificans Δ*tsdA shdrR-Cys^{116}Ser*	Exchange of sHdrR-Cys^{116} to Ser in *H. denitrificans* Δ*tsdA*	This work
H. denitrificans Δ*tsdA shdrR- Cys^{50}Ser-Cys^{116}Ser*	Exchange of sHdrR- Cys^{50} and Cys^{116} to Ser in *H. denitrificans* Δ*tsdA*	This work
**Primers**		
Fr-pET22b-sHdrR-trun-NdeI	GGCA**CATATG**ACCGACGCGTCGATCGAACAG (NdeI)	This work
Rev-pET22b-0682-NotI	TTTT**GCGGCCGC**ATTCGAGCGTTTTCCCGCAC (NotI)	(Li et al., 2023b)
sHdrR _C50S_Up_Rev	GGTTCTTTCTCCCTCGAGCAGGAGGGACAAAATCGCGAGA	This work
sHdrR _C50S_Down_Fw	TCTCGCGATTTTGTCCCTCCTGCTCGAGGGAGAAAGAACC	This work
sHdrR _C116S _Up_rev	GTTTTCCCGCACTCGTTGCACTATAATACTTATGCAGCGT	This work
sHdrR_C116S_Down_Fw	ACGCTGCATAAGTATTATAGTGCAACGAGTGCGGGAAAAC	This work
Fwd_deltaHden0682_BamHI	GCAT**GGATCC**GCGAAAATGTGCACCGGAG (BamHI)	(Li et al., 2023b)
Rev_deltaHden0682_XbaI	AAGC**TCTAGA**TATGCGGCAGCCGTTGACGC (XbaI)	(Li et al., 2023b)
EMSA-Fr	TTCCCGCCCCGTCTTGGTTT	(Li et al., 2023b)
EMSA_Fr2_Fr	TCAGCGCTCGCCTGGAAGTC	(Li et al., 2024)
EMSA_Fr3_Rev	TCTAAGCATCAACATATTCATATCTTTATATATTTTCG	(Li et al., 2024)
EMSA-Rev	AGGAGTTGCATCCAAAAAAGCGTG	(Li et al., 2023b)
EMSA-Hden_0703/04-fw	GGGTCACCAAATTCTGCAGGTCTC	(Li et al., 2024)
EMSA-Hden_0703/04-rev	ATCACGCCATCTCTCCCGGAA	(Li et al., 2024)
EMSA-Hden_0699/0698-fw	AATTCCACGGCTCCGCC	(Li et al., 2024)
EMSA-Hden_0699/0698-rev	TCGACAGCTTGCGGAAATCC	(Li et al., 2024)
EMSA-sHdrR-LipS1_F	TAGAGCGAGTCTTCAGC	(Li et al., 2024)
EMSA-sHdrR-LipS1_R	CGGCCCTCTGAGAAAAG	(Li et al., 2024)
EMSA-LipX-DsrE_F	GACTTCGCCGATCAATCGATC	(Li et al., 2024)
EMSA-LipX-DsrE_R	TGCCACCTCCCCGATATG	(Li et al., 2024)
EMSA-Hden_0703/04-fw	GGGTCACCAAATTCTGCAGGTCTC	(Li et al., 2024)
rpoB-denitf	AGGACGTGTTCACCTCGATT	(Martineau et al., 2015)
rpoB-denitr	CGGCTTCGTCAAGGTTCTTC	(Martineau et al., 2015)
SoxT1A 0681_qPCR-Fr	CCCGAGTGATACGATTCGCA	(Li et al., 2023a)
SoxT1A 0681_qPCR-Rev	CTAAAATGCCGCCGGTGATG	(Li et al., 2023a)
LplA_qPCR-Fr	GGCCATGATCGATTTGCACC	(Li et al., 2024)
LplA_qPCR-Rev	CGAGATAAATTGCACCGCCG	(Li et al., 2024)
sHdrA_qPCR-Fr	CCGATCACCATTCCGTTCGA	(Li et al., 2023a)
sHdrA_qPCR-Rev	CAATTGTTTCCGGGCCGATC	(Li et al., 2023a)
sHdrB2_qPCR-Fr	GACGTGGCCTACTATTCCGG	(Li et al., 2024)
sHdrB2_qPCR-Rev	CCGCGACGACAGATAGGTTT	(Li et al., 2024)
LbpA2_qPCR-Fr	GGTTCCAAGAGCAGCCTGAT	(Li et al., 2024)
LbpA2_qPCR-Rev	TCGTTGATCTCCAGAACCGC	(Li et al., 2024)
SoxXA_qPCR-Fr	CGGCGCTCATTACCTATCTC	(Li et al., 2024)

SoxXA_qPCR-Rev	TCGGGGTGTCTTTTTCAGTC	(Li et al., 2024)
TusA_qPCR-Fr	TCTGACAGTTGATGCCAAGG	(Li et al., 2024)
TusA_qPCR-Rev	CGTTTCCTCATGTTCAAGCA	(Li et al., 2024)
CytP450_qPCR-Fr	CAATACGGTTCTCGGACGTT	(Li et al., 2024)
CytP450_qPCR-Rev	CATTCGTTTCCTGACGAGGT	(Li et al., 2024)
SoxT1B (0699)_qPCR-Fr	GCCGCCGTCTCAGTAAATAA	(Li et al., 2024)
SoxT1B (0699)_qPCR-Rev	AGCAGAAGACGGCAGATGAT	(Li et al., 2024)
SoxR_qPCR-Fr	TGAAGCGGACGAGGAAGTAT	(Li et al., 2024)
SoxR_qPCR-Rev	GAGACTGTGGGCTGGTTGAT	(Li et al., 2024)
sHdrR_qPCR-Fr	TTAGGAAGTCCGCATCGTCT	(Li et al., 2024)
sHdrR_qPCR-Rev	GCACTCGTTGCGCAATAATA	(Li et al., 2024)
SoxY_qPCR-Fr	GTTCAGCTTGCGGACTTTTC	(Li et al., 2024)
SoxY_qPCR-Rev	GCCAATCGTCACCTTCACTT	(Li et al., 2024)
**Plasmids**		
pHP45Ω-Tc	Ap^r, Tc^r	(Fellay et al., 1987)
pk18*mobsacB*	Km^r, Mob$^+$, *sacB*, *oriV*, *oriT*, *lacZα*	(Schäfer et al., 1994)
pET-22b (+)	ApR, T7 promoter, lac operator, C-terminal His tag, pelB leader	Novagen
pET-22bHdsHdrR-trunc	Ap^R, NdeI-NotI fragment of PCR amplified truncated *shdrR* in NdeI-NotI of p ET-22b (+)	This work
pk18*mobsacB-shdrR*	Km^r, 2379 bp PCR fragment for chromosomal complementation of *shdrR* cloned int pk18*mobsacB* using XbaI and BamHI sites	This work
pk18*mobsacB-shdrR*-Tc	Km^r, Tc^r, pHP45ΩTc tetracycline cassette inserted into pk18*mobsacB-shdrR* using SmaI	This work
pk18*mobsacB-shdrR-C50S*	Km^r, SOE PCR fragment implementing chromosomal integration of *shdrR* encoding a Cys^{50}Ser exchange cloned into pk18*mobsacB* using XbaI and BamHI restriction sites	This work
pk18*mobsacB-shdrR-C50S*-Tc	Km^r, Tc^r, pHP45ΩTc tetracycline cassette inserted into pk18*mobsacB-shdrR-C50S* using SmaI	This work
pk18*mobsacB-shdrR-C116S*	Km^r, SOE PCR fragment implementing chromosomal integration of *shdrR* encoding a Cys^{116}Ser exchange cloned into pk18*mobsacB* using XbaI and BamHI restriction sites	This work
pk18*mobsacB-shdrR-C116S*-Tc	Km^r, Tc^r, pHP45ΩTc tetracycline cassette inserted into pk18*mobsacB-shdrR-C116S* using SmaI	This work
pk18*mobsacB-shdrR-C50S-C116S*	Km^r, SOE PCR fragment implementing chromosomal integration of *shdrR* encoding Cys^{50}Ser and Cys^{116}Ser exchange cloned into pk18*mobsacB* using XbaI and BamHI restriction sites	This work
pk18*mobsacB-shdrR-C50S-C116S*-Tc	Km^r, Tc^r, pHP45ΩTc tetracycline cassette inserted into pk18*mobsacB-shdrR-C50S-C116S* using SmaI	This work

Supplementary Table 5. RNA-Seq quality control

Part a. Read statistics with sequence quality metrics overview. For each sample, the following metrics are provided: sample name, total raw reads, total high quality (HQ) reads after sequence cleaning and filtering (Chen et al., 2018), percentage of high quality (HQ) bases having at least Phred quality 30, GC content in percentile of high quality sequencing reads, mean read length (bp), high quality reads percentage.

Sample Name	Total Raw Reads	Total HQ Reads	HQ Bases (Q30)	GC Content	Mean Read Length (bp)	HQ Reads %
deltasHdrR_a	13.5 M	13.48 M	93.16%	59.15%	149	99.84%
deltasHdrR_b	13.5 M	13.48 M	93.75%	59.13%	149	99.87%
deltaSoxR_a	13.5 M	13.48 M	92.98%	59.75%	150	99.85%
deltaSoxR_b	13.5 M	13.47 M	93.50%	59.49%	149	99.80%
deltaTsdA_a	13.5 M	13.42 M	93.37%	59.56%	149	99.41%
deltaTsdA_b	13.5 M	13.48 M	93.19%	59.94%	149	99.86%
deltaTsdATs_a	13.5 M	13.47 M	93.41%	59.49%	149	99.81%
deltaTsdATs_b	13.5 M	13.43 M	93.59%	59.74%	150	99.49%

Part b. Alignment statistics. For each sample, the following statistics are provided: sample name, total raw ready, total high quality (HQ) reads after sequence cleaning and filtering (Chen et al., 2018), the total number of reads mapped to the reference genome, the number of reads that could not get mapped to the reference, number of reads that can only be mapped to one reference locus

Sample	Total HQ Reads	Mapped Reads	Unmapped Reads	Unique Reads
deltaTsdA_a	13.42M	12.32M (91.82%)	1.10M (8.18%)	12.27M (91.46%)
deltaTsdA_b	13.48M	12.39M (91.93%)	1.09M (8.07%)	12.35M (91.59%)
deltaTsdATs_	13.47M	12.60M (93.52%)	873.06K (6.48%)	12.55M (93.14%)
deltaTsdATs_b	13.43M	12.57M (93.56%)	864.77K (6.44%)	12.52M (93.20%)
deltasHdrR_	13.48M	12.28M (91.09%)	1.20M (8.91%)	12.22M (90.69%)
deltasHdrR_b	13.48M	12.28M (91.06%)	1.21M (8.94%)	12.22M (90.66%)
deltaSoxR_a	13.48M	13.04M (96.72%)	442.52K (3.28%)	12.98M (96.30%)
deltaSoxR_b	13.47M	13.03M (96.69%)	446.26K (3.31%)	12.97M (96.27%)

References

Appia-Ayme, C., Little, P.J., Matsumoto, Y., Leech, A.P., and Berks, B.C. (2001) Cytochrome complex essential for photosynthetic oxidation of both thiosulfate and sulfide in *Rhodovulum sulfidophilum*. *Journal of Bacteriology* **183**: 6107-6118.

Barbosa, R.L., and Benedetti, C.E. (2007) BigR, a transcriptional repressor from plant-associated bacteria, regulates an operon implicated in biofilm growth. *Journal of Bacteriology* **189**: 6185-6194.

Beller, H.R., Chai, P.S.G., Letain, T.E., Chakicherla, A., Larimer, F.W., Richardson, P.M., Coleman, M.A., Wood, A.P., and Kelly, D.P. (2006) The genome sequence of the obligately chemolithoautotrophic, facultatively anaerobic bacterium *Thiobacillus denitrificans*. *Journal of Bacteriology* **188**: 1473-1488.

Brazilian National Genome Project, C. (2003) The complete genome sequence of *Chromobacterium violaceum* reveals remarkable and exploitable bacterial adaptability. *Proceedings of the National Academy of Sciences of the United States of America* **100**: 11660-11665.

Capdevila, D.A., Walsh, B.J.C., Zhang, Y., Dietrich, C., Gonzalez-Gutierrez, G., and Giedroc, D.P. (2021) Structural basis for persulfide-sensing specificity in a transcriptional regulator. *Nature Chemical Biology* **17**: 65-70.

Challacombe, J.F., Majid, S., Deole, R., Brettin, T.S., Bruce, D., Delano, S.F., Detter, J.C., Gleasner, C.D., Han, C.S., Misra, M., Reitenga, K.G., Mikhailova, N., Woyke, T., Pitluck, S., Nolan, M., Land, M.L., Saunders, E., Tapia, R., Lapidus, A., Ivanova, N., and Hoff, W.D. (2013) Complete genome sequence of *Halorhodospira halophila* SL1. *Standards in Genomic Sciences* **8**: 206-214.

Chen, S., Zhou, Y., Chen, Y., and Gu, J. (2018) fastp: an ultra-fast all-in-one FASTQ preprocessor. *Bioinformatics* **34**: i884-i890.

Chewapreecha, C., Harris, S.R., Croucher, N.J., Turner, C., Marttinen, P., Cheng, L., Pessia, A., Aanensen, D.M., Mather, A.E., Page, A.J., Salter, S.J., Harris, D., Nosten, F., Goldblatt, D., Corander, J., Parkhill, J., Turner, P., and Bentley, S.D. (2014) Dense genomic sampling identifies highways of pneumococcal recombination. *Nature Genetics* **46**: 305-309.

Crombie, A.T., Larke-Mejia, N.L., Emery, H., Dawson, R., Pratscher, J., Murphy, G.P., McGenity, T.J., and Murrell, J.C. (2018) Poplar phyllosphere harbors disparate isoprene-degrading bacteria. *Proceedings of the National Academy of Sciences of the United States of America* **115**: 13081-13086.

Dai, W., Zhu, Y., Wang, X., Sakenova, N., Yang, Z., Wang, H., Li, G., He, J., Huang, D., Cai, Y., Guo, W., Wang, Q., Feng, T., Fan, Q., Zheng, T., and Han, A. (2016) Draft genome sequence of the bacterium *Comamonas aquatica* CJG. *Genome Announcements* **4**: e01186-01116.

Fellay, R., Frey, J., and Krisch, H.M. (1987) Interposon mutagenesis of soil and water bacteria: a family of DNA fragments designed for in vitro insertional mutagenesis of Gram-negative bacteria. *Gene* **52**: 147-154.

Goodner, B., Hinkle, G., Gattung, S., Miller, N., Blanchard, M., Qurollo, B., Goldman, B.S., Cao, Y., Askenazi, M., Halling, C., Mullin, L., Houmiel, K., Gordon, J., Vaudin, M., Iartchouk, O., Epp, A., Liu, F., Wollam, C., Allinger, M., Doughty, D., Scott, C., Lappas, C., Markelz, B., Flanagan, C., Crowell, C., Gurson, J., Lomo, C., Sear, C., Strub, G., Cielo, C., and Slater, S. (2001) Genome sequence of the plant pathogen and biotechnology agent *Agrobacterium tumefaciens* C58. *Science* **294**: 2323-2328.

Gristwood, T., McNeil, M.B., Clulow, J.S., Salmond, G.P., and Fineran, P.C. (2011) PigS and PigP regulate prodigiosin biosynthesis in *Serratia* via differential control of divergent operons, which include predicted transporters of sulfur-containing molecules. *Journal of Bacteriology* **193**: 1076-1085.

Gueuné, H., Durand, M.J., Thouand, G., and DuBow, M.S. (2008) The *ygaVP* genes of *Escherichia coli* form a tributyltin-inducible operon. *Applied and Environmental Microbiology* **74**: 1954-1958.

Guimarães, B.G., Barbosa, R.L., Soprano, A.S., Campos, B.M., de Souza, T.A., Tonoli, C.C., Leme, A.F., Murakami, M.T., and Benedetti, C.E. (2011) Plant pathogenic bacteria utilize biofilm growth-associated repressor (BigR), a novel winged-helix redox switch, to control hydrogen sulfide detoxification under hypoxia. *Journal of Biological Chemistry* **286**: 26148-26157.

He, C., Keren, R., Whittaker, M.L., Farag, I.F., Doudna, J.A., Cate, J.H.D., and Banfield, J.F. (2021) Genome-resolved metagenomics reveals site-specific diversity of episymbiotic CPR bacteria and DPANN archaea in groundwater ecosystems. *Nature Microbiology* **6**: 354-365.

Heidelberg, J.F., Eisen, J.A., Nelson, W.C., Clayton, R.A., Gwinn, M.L., Dodson, R.J., Haft, D.H., Hickey, E.K., Peterson, J.D., Umayam, L., Gill, S.R., Nelson, K.E., Read, T.D., Tettelin, H., Richardson, D., Ermolaeva, M.D., Vamathevan, J., Bass, S., Qin, H., Dragoi, I., Sellers, P., McDonald, L., Utterback, T., Fleishmann, R.D., Nierman, W.C., White, O., Salzberg, S.L., Smith, H.O., Colwell, R.R., Mekalanos, J.J., Venter, J.C., and Fraser, C.M. (2000) DNA sequence of both chromosomes of the cholera pathogen *Vibrio cholerae*. *Nature* **406**: 477-483.

Hoang, D.T., Chernomor, O., von Haeseler, A., Minh, B.Q., and Vinh, L.S. (2018) UFBoot2: Improving the ultrafast bootstrap approximation. *Molecular Biology and Evolution* **35**: 518-522.

Hong, S., Kim, J., Cho, E., Na, S., Yoo, Y.J., Cho, Y.H., Ryu, S., and Ha, N.C. (2022) Crystal structures of YeiE from *Cronobacter sakazakii* and the role of sulfite tolerance in gram-negative bacteria. *Proceedings of the National Academy of Sciences of the United States of America* **119**: e2118002119.

Imhoff, J.F., Rahn, T., Kunzel, S., and Neulinger, S.C. (2018) New insights into the metabolic potential of the phototrophic purple bacterium *Rhodopila globiformis* DSM 161^T from its draft genome sequence and evidence for a vanadium-dependent nitrogenase. *Archives of Microbiology* **200**: 847-857.

Jiang, Y.L., Wang, X.P., Sun, H., Han, S.J., Li, W.F., Cui, N., Lin, G.M., Zhang, J.Y., Cheng, W., Cao, D.D., Zhang, Z.Y., Zhang, C.C., Chen, Y., and Zhou, C.Z. (2018) Coordinating carbon and nitrogen metabolic signaling through the cyanobacterial global repressor NdhR. *Proceedings of the National Academy of Sciences of the United States of America* **115**: 403-408.

Kalhoefer, D., Thole, S., Voget, S., Lehmann, R., Liesegang, H., Wollher, A., Daniel, R., Simon, M., and Brinkhoff, T. (2011) Comparative genome analysis and genome-guided physiological analysis of *Roseobacter litoralis*. *BMC Genomics* **12**: 324.

Kaneko, T., Nakamura, Y., Sato, S., Minamisawa, K., Uchiumi, T., Sasamoto, S., Watanabe, A., Idesawa, K., Iriguchi, M., Kawashima, K., Kohara, M., Matsumoto, M., Shimpo, S., Tsuruoka, H., Wada, T., Yamada, M., and Tabata, S. (2002) Complete genomic sequence of nitrogen-fixing symbiotic bacterium *Bradyrhizobium japonicum* USDA110. *DNA Research* **9**: 189-197.

Kantor, R.S., van Zyl, A.W., van Hille, R.P., Thomas, B.C., Harrison, S.T., and Banfield, J.F. (2015) Bioreactor microbial ecosystems for thiocyanate and cyanide degradation unravelled with genome-resolved metagenomics. *Environmental Microbiology* **17**: 4929-4941.

Koblizek, M., Janouskovec, J., Obornik, M., Johnson, J.H., Ferriera, S., and Falkowski, P.G. (2011) Genome sequence of the marine photoheterotrophic bacterium *Erythrobacter* sp. strain NAP1. *Journal of Bacteriology* **193**: 5881-5882.

Koch, T., and Dahl, C. (2018) A novel bacterial sulfur oxidation pathway provides a new link between the cycles of organic and inorganic sulfur compounds. *ISME Journal* **12**: 2479-2491.

Kümpel, C., Grosser, M., Tanabe, T.S., and Dahl, C. (2024) Fe/S proteins in microbial sulfur oxidation. *Biochimica et Biophysica Acta (BBA) - Molecular Cell Research* **1871**: 119732.

Lacher, D.W., Mammel, M.K., Gangiredla, J., Gebru, S.T., Barnaba, T.J., Majowicz, S.A., and Dudley, E.G. (2020) Draft genome sequences of isolates of diverse host origin from the *E. coli* Reference Center at Penn State University. *Microbiology Resource Announcements* **9**.

Larimer, F.W., Chain, P., Hauser, L., Lamerdin, J., Malfatti, S., Do, L., Land, M.L., Pelletier, D.A., Beatty, J.T., Lang, A.S., Tabita, F.R., Gibson, J.L., Hanson, T.E., Bobst, C., Torres y Torres, J.L., Peres, C., Harrison, F.H., Gibson, J., and Harwood, C.S. (2004) Complete genome sequence of the metabolically versatile photosynthetic bacterium *Rhodopseudomonas palustris*. *Nature Biotechnology* **22**: 55-61.

LaSarre, B., Kysela, D.T., Stein, B.D., Ducret, A., Brun, Y.V., and McKinlay, J.B. (2018) Restricted localization of photosynthetic intracytoplasmic membranes (ICMs) in multiple genera of purple nonsulfur bacteria. *Mbio* **9**.

Li, J., Törkel, K., Koch, J., Tanabe, T.S., Hsu, H.Y., and Dahl, C. (2023a) In the Alphaproteobacterium *Hyphomicrobium denitrificans* SoxR serves as a sulfane sulfur-responsive repressor of sulfur oxidation. *Antioxidants* **12**: 1620.

Li, J., Koch, J., Flegler, W., Garcia Ruiz, L., Hager, N., Ballas, A., Tanabe, T.S., and Dahl, C. (2023b) A metabolic puzzle: consumption of C_1 compounds and thiosulfate in *Hyphomicrobium denitrificans* X^T. *Biochimica et Biophysica Acta (BBA) - Bioenergetics* **1864**: 148932.

Li, J., Göbel, F., Hsu, H.Y., Koch, J.N., Hager, N., Flegler, W., Tanabe, T.S., and Dahl, C. (2024) YeeE-like bacterial SoxT proteins mediate sulfur transport for oxidation and signal transduction. *Communications Biology* **7**: 1548.

Mandal, S., Chatterjee, S., Dam, B., Roy, P., and Das Gupta, S.K. (2007) The dimeric repressor SoxR binds cooperatively to the promoter(s) regulating expression of the sulfur oxidation (*sox*) operon of *Pseudaminobacter salicylatoxidans* KCT001. *Microbiology* **153**: 80-91.

Martineau, C., Mauffrey, F., and Villemur, R. (2015) Comparative analysis of denitrifying activities of *Hyphomicrobium nitrativorans*, *Hyphomicrobium denitrificans*, and *Hyphomicrobium zavarzinii*. *Applied and Environmental Microbiology* **81**: 5003-5014.

Minh, B.Q., Schmidt, H.A., Chernomor, O., Schrempf, D., Woodhams, M.D., von Haeseler, A., and Lanfear, R. (2020) IQ-TREE 2: New models and efficient methods for phylogenetic inference in the genomic era. *Molecular Biology and Evolution* **37**: 1530-1534.

Mukherjee, D., Datta, A.B., and Chakrabarti, P. (2014) Crystal structure of HlyU, the hemolysin gene transcription activator, from *Vibrio cholerae* N16961 and functional implications. *Biochimica Biophysica Acta* **1844**: 2346-2354.

Munk, A.C., Copeland, A., Lucas, S., Lapidus, A., Del Rio, T.G., Barry, K., Detter, J.C., Hammon, N., Israni, S., Pitluck, S., Brettin, T., Bruce, D., Han, C., Tapia, R., Gilna, P., Schmutz, J., Larimer, F., Land, M., Kyrpides, N.C., Mavromatis,

K., Richardson, P., Rohde, M., Goker, M., Klenk, H.P., Zhang, Y., Roberts, G.P., Reslewic, S., and Schwartz, D.C. (2011) Complete genome sequence of *Rhodospirillum rubrum* type strain (S1). *Standards in Genomic Sciences* **4**: 293-302.

Nikaido, H. (2011) Structure and mechanism of RND-type multidrug efflux pumps. *Advances in Enzymology and Related Areas of Molecular Biology* **77**: 1-60.

Paul, K.B., and Larson, T.J. (2006) ArsR homolog YgaV autoregulates the theorized ygaV - ygaP rhodanese operon in *Escherichia coli*. *FASEB Journal* **20**: A70.

Pis Diez, C.M., Antelo, G.T., Dalia, T.N., Dalia, A.B., Giedroc, D.P., and Capdevila, D.A. (2023) Increased intracellular persulfide levels attenuate HlyU-mediated hemolysin transcriptional activation in *Vibrio cholerae*. *Journal of Biological Chemistry* **299**: 105147.

Riley, M., Abe, T., Arnaud, M.B., Berlyn, M.K., Blattner, F.R., Chaudhuri, R.R., Glasner, J.D., Horiuchi, T., Keseler, I.M., Kosuge, T., Mori, H., Perna, N.T., Plunkett, G., 3rd, Rudd, K.E., Serres, M.H., Thomas, G.H., Thomson, N.R., Wishart, D., and Wanner, B.L. (2006) *Escherichia coli* K-12: a cooperatively developed annotation snapshot--2005. *Nucleic Acids Research* **34**: 1-9.

Rother, D., Orawski, G., Bardischewsky, F., and Friedrich, C.G. (2005) SoxRS-mediated regulation of chemotrophic sulfur oxidation in *Paracoccus pantotrophus*. *Microbiology* **151**: 1707-1716.

Schäfer, A., Tauch, A., Jäger, W., Kalinowski, J., Thierbach, G., and Pühler, A. (1994) Small mobilizable multi-purpose cloning vectors derived from the *Escherichia coli* plasmids pK18 and pK19: selection of defined deletions in the chromosome of *Corynebacterium glutamicum*. *Gene* **145**: 69-73.

Shimizu, T., Shen, J., Fang, M., Zhang, Y., Hori, K., Trinidad, J.C., Bauer, C.E., Giedroc, D.P., and Masuda, S. (2017) Sulfide-responsive transcriptional repressor SqrR functions as a master regulator of sulfide-dependent photosynthesis. *Proceedings of the National Academy of Sciences of the United States of America* **114**: 2355-2360.

Simpson, A.J., Reinach, F.C., Arruda, P., Abreu, F.A., Acencio, M., Alvarenga, R., Alves, L.M., Araya, J.E., Baia, G.S., Baptista, C.S., Barros, M.H., Bonaccorsi, E.D., Bordin, S., Bove, J.M., Briones, M.R., Bueno, M.R., Camargo, A.A., Camargo, L.E., Carraro, D.M., Carrer, H., Colauto, N.B., Colombo, C., Costa, F.F., Costa, M.C., Costa-Neto, C.M., Coutinho, L.L., Cristofani, M., Dias-Neto, E., Docena, C., El-Dorry, H., Facincani, A.P., Ferreira, A.J., Ferreira, V.C., Ferro, J.A., Fraga, J.S., Franca, S.C., Franco, M.C., Frohme, M., Furlan, L.R., Garnier, M., Goldman, G.H., Goldman, M.H., Gomes, S.L., Gruber, A., Ho, P.L., Hoheisel, J.D., Junqueira, M.L., Kemper, E.L., Kitajima, J.P., Krieger, J.E., Kuramae, E.E., Laigret, F., Lambais, M.R., Leite, L.C., Lemos, E.G., Lemos, M.V., Lopes, S.A., Lopes, C.R., Machado, J.A., Machado, M.A., Madeira, A.M., Madeira, H.M., Marino, C.L., Marques, M.V., Martins, E.A., Martins, E.M., Matsukuma, A.Y., Menck, C.F., Miracca, E.C., Miyaki, C.Y., Monteriro-Vitorello, C.B., Moon, D.H., Nagai, M.A., Nascimento, A.L., Netto, L.E., Nhani, A., Jr., Nobrega, F.G., Nunes, L.R., Oliveira, M.A., de Oliveira, M.C., de Oliveira, R.C., Palmieri, D.A., Paris, A., Peixoto, B.R., Pereira, G.A., Pereira, H.A., Jr., Pesquero, J.B., Quaggio, R.B., Roberto, P.G., Rodrigues, V., de, M.R.A.J., de Rosa, V.E., Jr., de Sa, R.G., Santelli, R.V., Sawasaki, H.E., da Silva, A.C., da Silva, A.M., da Silva, F.R., da Silva, W.A., Jr., da Silveira, J.F., Silvestri, M.L., Siqueira, W.J., de Souza, A.A., de Souza, A.P., Terenzi, M.F., Truffi, D., Tsai, S.M., Tsuhako, M.H., Vallada, H., Van Sluys, M.A., Verjovski-Almeida, S., Vettore, A.L., Zago, M.A., Zatz, M., Meidanis, J., and Setubal, J.C. (2000) The genome sequence of the plant pathogen *Xylella fastidiosa*. The Xylella fastidiosa Consortium of the Organization for Nucleotide Sequencing and Analysis. *Nature* **406**: 151-159.

Tanabe, T.S., and Dahl, C. (2023) HMSS2: an advanced tool for the analysis of sulfur metabolism, including organosulfur compound transformation, in genome and metagenome assemblies. *Molecular Ecology Resources* **23**: 1930-1945.

Tatusova, T., Ciufo, S., Fedorov, B., O'Neill, K., and Tolstoy, I. (2014) RefSeq microbial genomes database: new representation and annotation strategy. *Nucleic Acids Research* **42**: D553-D559.

Thöny-Meyer, L. (2002) Cytochrome *c* maturation: a complex pathway for a simple task? *Biochemical Society Transactions* **30**: 633-638.

Trifinopoulos, J., Nguyen, L.T., von Haeseler, A., and Minh, B.Q. (2016) W-IQ-TREE: a fast online phylogenetic tool for maximum likelihood analysis. *Nucleic Acids Research* **44**: W232-235.

Valdes, J., Ossandon, F., Quatrini, R., Dopson, M., and Holmes, D.S. (2011) Draft genome sequence of the extremely acidophilic biomining bacterium *Acidithiobacillus thiooxidans* ATCC 19377 provides insights into the evolution of the *Acidithiobacillu*s genus. *Journal of Bacteriology* **193**: 7003-7004.

Venkatramanan, R., Prakash, O., Woyke, T., Chain, P., Goodwin, L.A., Watson, D., Brooks, S., Kostka, J.E., and Green, S.J. (2013) Genome sequences for three denitrifying bacterial strains isolated from a uranium- and nitrate-contaminated subsurface environment. *Genome Announcements* **1**.

Weinitschke, S., Denger, K., Cook, A.M., and Smits, T.H.M. (2007) The DUF81 protein TauE in *Cupriavidus necator* H16, a sulfite exporter in the metabolism of C_2 sulfonates. *Microbiology* **153**: 3055-3060.

Weissgerber, T., Zigann, R., Bruce, D., Chang, Y.J., Detter, J.C., Han, C., Hauser, L., Jeffries, C.D., Land, M., Munk, A.C., Tapia, R., and Dahl, C. (2011) Complete genome sequence of *Allochromatium vinosum* DSM 180^{T}. *Standards in Genomic Sciences* **5**: 311-330.

Williams, S.G., Attridge, S.R., and Manning, P.A. (1993) The transcriptional activator HlyU of *Vibrio cholerae*: nucleotide sequence and role in virulence gene expression. *Molecular Microbiology* **9**: 751-760.

Wodara, C., Bardischewsky, F., and Friedrich, C.G. (1997) Cloning and characterization of sulfite dehydrogenase, two *c*-type cytochromes, and a flavoprotein of *Paracoccus denitrificans* GB17: essential role of sulfite dehydrogenase in lithotrophic sulfur oxidation. *Journal of Bacteriology* **179**: 5014-5023.

Wu, X.L., Yu, S.L., Gu, J., Zhao, G.F., and Chi, C.Q. (2009) *Filomicrobium insigne* sp. nov., isolated from an oil-polluted saline soil. *International Journal of Systematic and Evolutionary Microbiology* **59**: 300-305.

Zhang, Y., Kitajima, M., Whittle, A.J., and Liu, W.T. (2017) Benefits of genomic insights and CRISPR-Cas signatures to monitor potential pathogens across drinking water production and distribution systems. *Frontiers in Microbiology* **8**: 2036.
